# Interface Modification for Energy Level Alignment and Charge Extraction in CsPbI_3_ Perovskite Solar Cells

**DOI:** 10.1021/acsenergylett.3c01522

**Published:** 2023-09-22

**Authors:** Zafar Iqbal, Fengshuo Zu, Artem Musiienko, Emilio Gutierrez-Partida, Hans Köbler, Thomas W. Gries, Gennaro V. Sannino, Laura Canil, Norbert Koch, Martin Stolterfoht, Dieter Neher, Michele Pavone, Ana Belen Muñoz-García, Antonio Abate, Qiong Wang

**Affiliations:** † Helmholtz-Zentrum Berlin für Materialien und Energie GmbH. Hahn-Meitner-Platz 1, 14109 Berlin, Germany; ‡ Institut für Physik & IRIS Adlershof, Humboldt-Universität zu Berlin, 12489 Berlin, Germany; § Institute for Physics and Astronomy, University of Potsdam, Karl-Liebknecht-Straße 24-25, 14476 Potsdam-Golm, Germany; ∥ The Chinese University of Hong Kong, Electronic Engineering Department, Shatin N.T., Hong Kong 999077, People’s Republic of China; ⊥ Department of Chemical Sciences, University of Naples Federico II, Comp. Univ. Monte S. Angelo, Via Cintia 26, 80126 Naples, Italy; # Department of Physics “Ettore Pancini”, University of Naples Federico II, Comp. Univ. Monte S. Angelo, via Cintia 26, 80126 Naples, Italy; ∇ Department of Chemistry, Bielefeld University, Universitätsstraße 25, 33615 Bielefeld, Germany; ○ Department of Chemical Materials and Production Engineering, University of Naples Federico II, Piazzale Vincenzo Tecchio 80, 80125 Naples, Italy

## Abstract

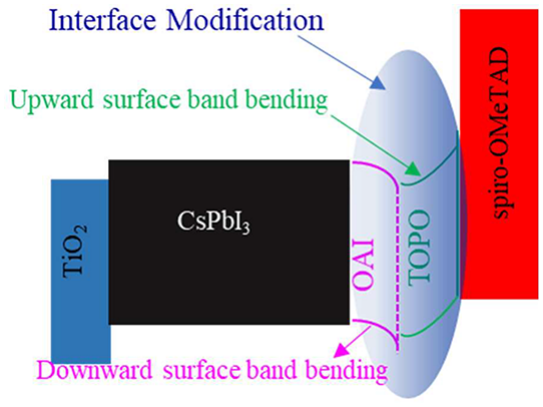

In perovskite solar cells (PSCs) energy level alignment and charge extraction at the interfaces are the essential factors directly affecting the device performance. In this work, we present a modified interface between all-inorganic CsPbI_3_ perovskite and its hole-selective contact (spiro-OMeTAD), realized by the dipole molecule trioctylphosphine oxide (TOPO), to align the energy levels. On a passivated perovskite film, with *n*-octylammonium iodide (OAI), we created an upward surface band-bending at the interface by TOPO treatment. This improved interface by the dipole molecule induces a better energy level alignment and enhances the charge extraction of holes from the perovskite layer to the hole transport material. Consequently, a *V*_oc_ of 1.2 V and a high-power conversion efficiency (PCE) of over 19% were achieved for inorganic CsPbI_3_ perovskite solar cells. Further, to demonstrate the effect of the TOPO dipole molecule, we present a layer-by-layer charge extraction study by a transient surface photovoltage (trSPV) technique accomplished by a charge transport simulation.

Since Miyasaka et al. reported halide perovskites for the first time as a light absorber in solar cells in 2009,^1^ the research community has improved the devices’ PCE to 26%,^2^ which makes perovskite solar cells (PSCs) potentially competitive with established technologies.^3,4^ However, the stability of PSCs is one of the Achilles heels to find the marketplace.^5,6^ The inorganic perovskite CsPbI_3_ has attracted attention due to its superior thermal stability compared to organic–inorganic lead perovskites.^7^ In CsPbI_3_-based PSCs, phase stability at the operating temperature and the crystallization processes are the main challenges associated with device fabrication.^8,9^ Recently, organic precursors, e.g. hydrogen lead iodide “HPbI_3_” and dimethylammonium iodide (DMAI), assisted film growth, and molten salt additive based strategies have been adopted to address these challenges.^10−13^ Besides, for all compositions open circuit voltage loss (defined as *E*_g_ – *eV*_oc,_ where *E*_g_ is the optical band gap of the absorber, *e* is the elementary charge, and *V*_oc_ is the measured open circuit voltage of the device) is higher in inorganic perovskite solar cells than in organic–inorganic solar cells.^14^ To date, over 21% efficiency has been achieved so far for CsPbI_3_ with a *V*_oc_ of over 1.2 V and a minimum open circuit voltage deficit (*V*_loss_) of 0.46 V,^15^ and thereby inorganic perovskite solar cells have a large open circuit voltage deficit as compared to organic–inorganic halide perovskite solar cells with a minimum *V*_loss_ of ∼0.3 V.^16^ Currently, inorganic perovskite film post-treatment methods have been very effective in increasing open-circuit voltage values.^17−19^ However, the energy level misalignment between the inorganic perovskite layer and the charge-selective contacts requires interfacial engineering to resolve the open circuit voltage deficit.^14,20,21^

An inorganic perovskite has a flat band surface, and introducing a surface band bending has been proven to be favorable for charge extraction.^22,23^ Canil et al.^24^ systematically studied how the Fermi-level of perovskite with (Cs_0.05_[MA_0.15_FA_0.85_PbI_0.85_Br_0.15_]_0.95_) composition can be tuned by the treatment of dipole molecules on the perovskite surface. Nazeeruddin et al. reported surface band bending on perovskite, comprising the triple-cation composition, by perhydropoly(silazane) (R_3_Si-NH-SiR_3_) treatment of the perovskite film^25^ and found that efficient hole extraction is achieved due to surface band bending. Recently, Wang et al. introduced n-type surface band bending with propylamine hydrochloride (PACl) molecule treatment for CsPbI_3_ inverted devices.^23^ This surface band bending incited better charge extraction, and they have reported 20.17% state-of-the-art efficiency for inverted CsPbI_3_-based solar cells. These reports suggest that surface band bending induced passivation is an effective strategy to address the energy level alignment and charge extraction at the interfaces. Surface passivation of the perovskite has been reported systematically as one of the most effective strategies to enhance stability in PSCs.^26,27^ Further, surface passivation, through 2D perovskites deposited on the top of 3D perovskites, is being employed to achieve higher efficiency and stability.^28,29^

In this work, we used previously reported *n*-octylammonium iodide (OAI) passivation on CsPbI_3_ to establish a control device.^30^ However, we have revealed in this work that the passivation of OAI on CsPbI_3_ leads to a downward band bending at the surface, which makes a more n-type perovskite film. For better energy level alignment, we have introduced the dipole molecule trioctylphosphine oxide (TOPO) to induce a perovskite upward surface band bending on a well-passivated perovskite film. These molecules (OAI and TOPO) have been used as passivating layers for the perovskite separately, as summarized in Table S1. Here, we designed an interface by employing these molecules simultaneously. This improved interface sample is termed “with TOPO” (w/TOPO). TOPO treatment on the control film in n-i-p PSCs improved the charge selectivity 6-fold, causing a decrease in energy offset and optimizing the energy level alignment, significantly impacting the stability of state-of-the-art inorganic PSCs.

Generally, the higher efficiency and stability are mainly attributed to the suppression of nonradiative recombination induced by surface defects.^31,32^ Elucidating the role of the dipole molecule (TOPO) in the suppression of interface nonradiative recombination was challenging to segregate by photoluminescence (PL) methods due to quenching phenomena.^33,34^ Both charge extraction at the interfaces and charge trapping at the defect states can contribute to quenching.^35−37^ Here we developed a method based on time-resolved surface photovoltage^37^ and charge transport simulation to resolve both carrier extraction and nonradiative recombination phenomena. This method helps to characterize the interface charge dynamics and interface kinetics. We also demonstrate the impact of the interfacial energy level alignment and the consequently improved charge extraction on the stability of PSCs.

With a band gap of around 1.7 eV,^38^ CsPbI_3_ has great potential for application as a top cell in a tandem structure with silicon or a narrow-band-gap perovskite film as the bottom cell.^39,40^ CsPbI_3_ perovskite thin films were prepared by adopting the method reported in one recent work from Seok’s group.^30^ Experimental details for perovskite film preparation and device fabrication can be found in the Supporting Information (SI). A potential influence of TOPO treatment on the perovskite film was investigated by UV–vis absorption, scanning electron microscopy, Kelvin probe force microscopy (KPFM), and macroscopic Kelvin probe (KP). The TOPO treatment barely changes absorption spectra, film thickness, and roughness as detailed in Figures S1–S5 in the SI.

We first determined steady-state photoluminescence (PL) for neat films that are the control samples and treated with TOPO (w/TOPO) (Figure 1). The slight change in peak position is attributed to the electronic structure modulation near the band edge,^41,42^ while the narrow PL spectrum for TOPO-treated samples indicates the lowest defect density in the film.^43^ For TOPO-treated samples at varying concentrations of 5, 10, 15, and 20 mM, these exhibited similar photoluminescence quantum yield (PLQY) and thus a similar quasi-Fermi level splitting (QFLS) (Figure S6b). This indicates that TOPO treatment makes barely any contribution to defect passivation. Time-resolved photoluminescence (TrPL) spectra, given in Figure 1b, were fitted with the biexponential eq S16 and discussed in Figures S7 and S8in the SI. The fitted parameters for the TRPL spectra are presented in Table S2. The fitted lifetime, *t*_2_, referring to the nonradiative recombination, showed that all samples have a very similar value of around 3.5 μs. Thus, this further supports the steady-state PL data that TOPO treatment does not contribute noticeably to defect passivation.

**Figure 1:**
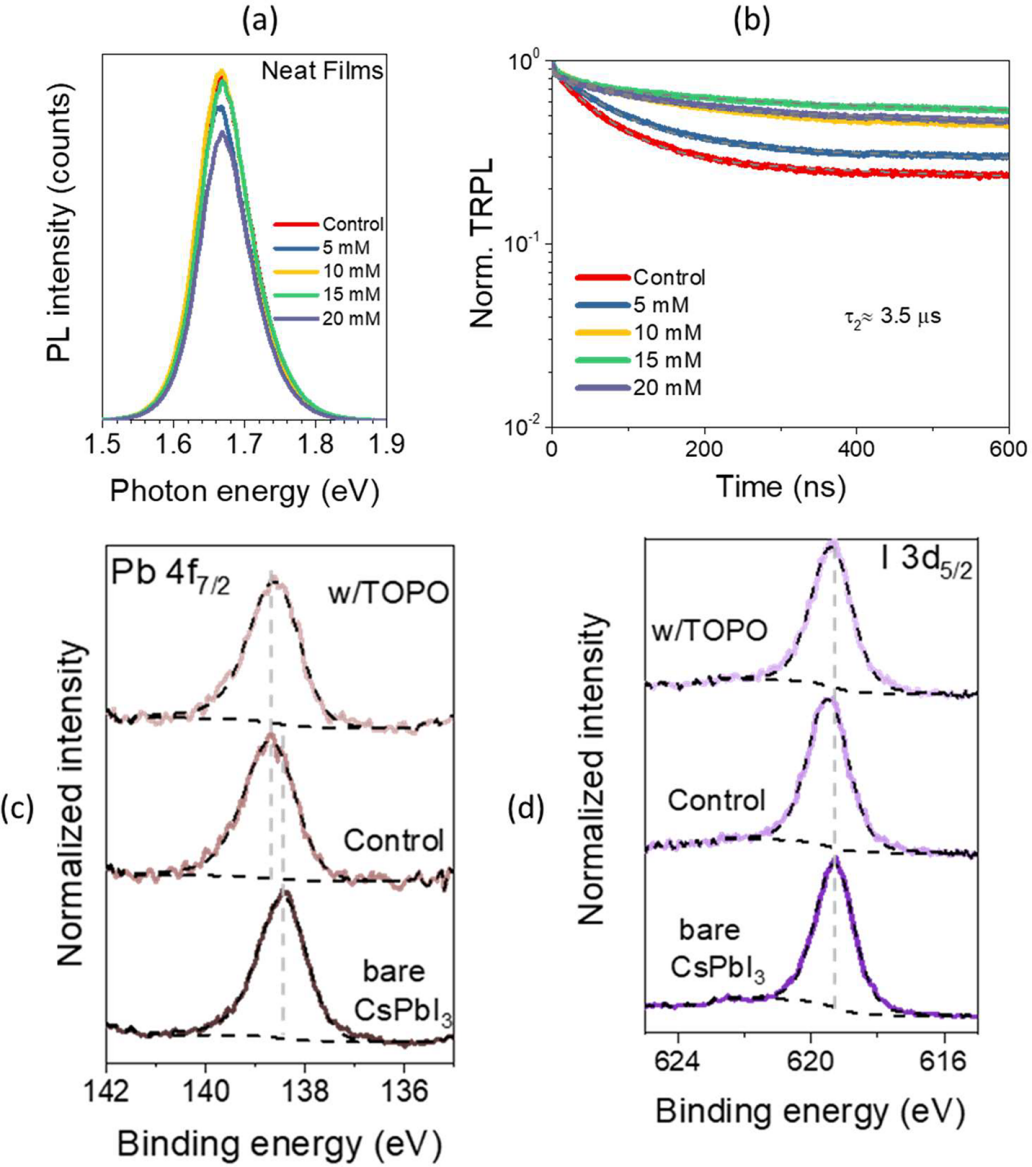
Surface passivation. (a) Steady-state and (b) time-resolved photoluminescence of neat perovskite films deposited on quartz for control (red line), 5 mM (steel blue line), 10 mM (yellow line), 15 mM (green line), and 20 mM (lavender line). TOPO treated samples at a fluence of XZ nJ cm^–2^ using an XZ nm laser. XPS of (c) Pb 4f_7/2_ and (d) I 3d_5/2_ core–shell spectra of bare CsPbI_3_, control sample, and w/TOPO. Dashed lines in black are fitted curves and backgrounds with guidelines in gray. The binding energy values for core–shell spectra are given in Figure S9.

X-ray photoelectron spectroscopy (XPS) results (Figure 1c,d) revealed that the OAI treatment shifted the Pb 4f and I 3d spectra toward higher binding energy by 250 and 200 meV, respectively. The values are detailed in Table S3. This is in line with a previous report and is likely due to the stronger coordination of the Pb–I bond with the introduction of a long-chain cation.^11^ The change in the work function position within the band gap can be determined from the shifts of occupied electronic states (valence and core levels). Analysis of core levels provides direct information on the change of the chemical states, but the valence bands are overlapped with contributions from perovskite and top layers, which render the analysis difficult. This is reflected as a less sharp valence onset in the valence spectra given in Figure 2. As a result, we estimated the work function change within the band gap, from the core level electron binding energy shift of I 3d (as it has a lower kinetic energy of the photoelectrons as compared to that of Pb 4f, which probes the magnitude of surface band bending more precisely), that leads to a downward band bending at the surface by 200 meV.

**Figure 2:**
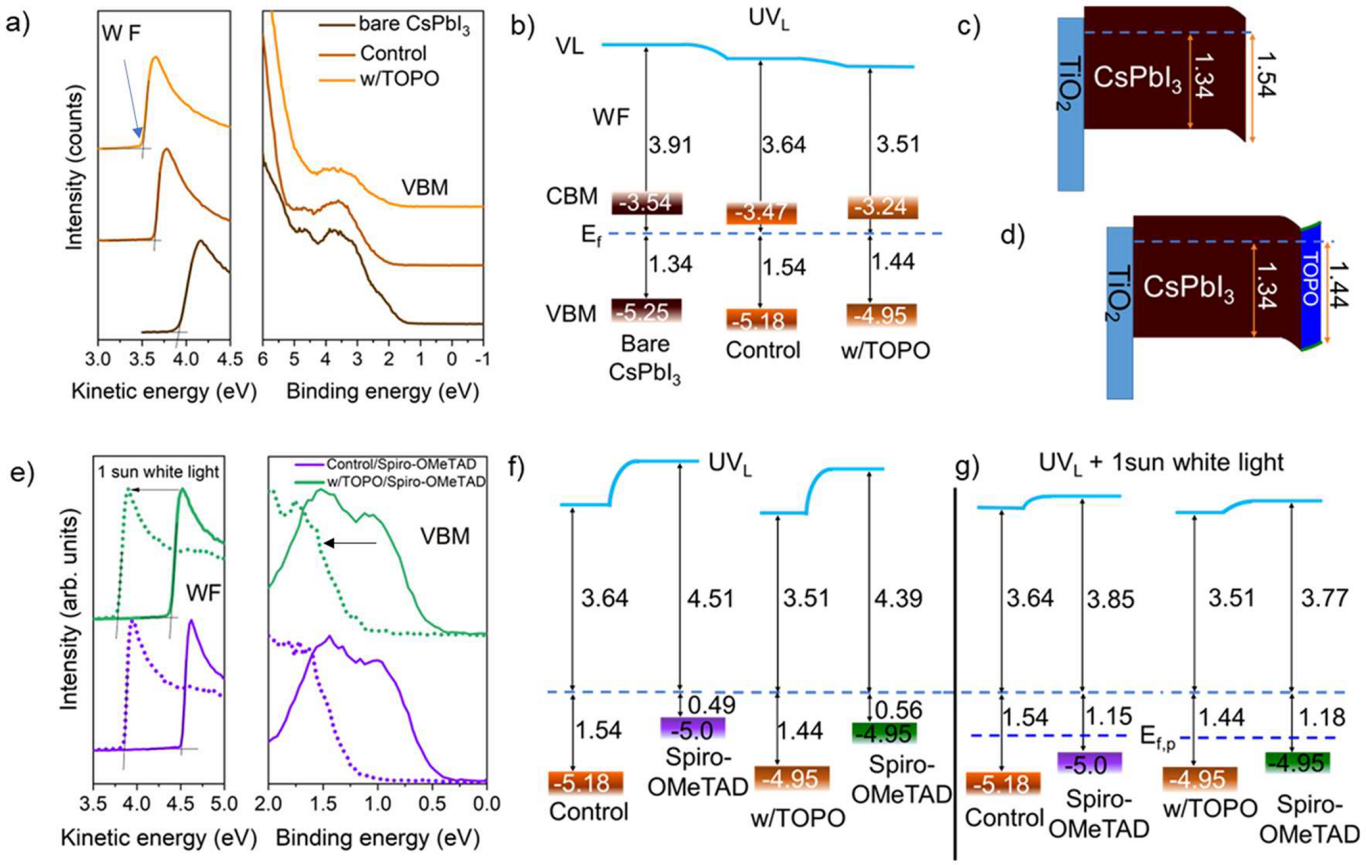
Energy level alignment. (a) Ultraviolet photoelectron spectroscopy (UPS) spectra of bare CsPbI_3_, control CsPbI_3_, and with TOPO treatment: secondary electron cutoff (SECO, left panel) and valence spectra (right panel). Energetic level scheme of (b) bare CsPbI_3_, control CsPbI_3_, and w/TOPO. Energy band diagram of (c) control perovskite and (d) with TOPO. (e) UPS spectra of control/spiro-OMeTAD, and w/TOPO/spiro-OMeTAD: SECO (left panel) and valence spectra (right panel). Dashed lines are UPS spectra measured in white light with 1 sun equivalent intensity. Energy band diagrams of control/spiro-OMeTAD and w/TOPO/spiro-OMeTAD (f) in the dark and (g) under 1 sun white light illumination. All samples were deposited on top of FTO/TiO_2_. UV_L_ refers to the low UV flux attenuated by the monochromator.

With further TOPO treatment, Pb 4f and I 3d peaks are shifted to lower binding energy by 80 and 100 meV, respectively. Thus, this indicates a lower downward band bending at the surface by 100 meV. We suggest here the core level binding energy shift in Pb 4f and I 3d comes from the electron-donating nature of TOPO:^44^ the ligand may donate electrons to positively charged ions, such as Pb^2+^ or iodine vacancies^45^ on the perovskite surface, thereby leading to a weaker coordination of the Pb–I bond. In addition, TOPO was reported to form a dipole layer at the surface of perovskite (as presented in Scheme S1) that points outward and reduces the work function (in other words, shifts the vacuum level to lower energy).^24,44^

The core level binding energy shift in Pb 4f and I 3d also implies direct contact between TOPO and the perovskite film in our work. This is reasonable, as we do not expect to have a compact thin layer of the OAI on top of the perovskite film that will lead to the formation of an insulating layer. Rather, OAI is expected to incorporate into the perovskite crystal structure and form a 2-dimensional structure on the surface.^29^ We suggest that OAI treatment has passivated most of the undercoordinated Pb^2+^ at the perovskite surface, and TOPO donates electrons more likely to the interfacial iodine vacancies as they are found to be benign.^45^ This would explain what we observed in PL analysis in Figure 1a that TOPO does not contribute noticeably to defect passivation. From the XPS in Figure S10, we detected signals for P 2s and O 1s peaks originating from TOPO molecules for TOPO-treated samples.

Figure 2a,e (left panel) shows the secondary electron cutoff spectra of all the samples, from which the work function (WF) can be obtained using the equation WF = *h*υ – *E*_cutoff_, where *h*υ is the photoelectron energy of He I light, and *E*_cutoff_ is the secondary electron cutoff. The right panel shows the valence band (VB) spectra of these samples on a linear intensity scale of the photoelectrons. It is noted that the VB onset of perovskites is, however, extrapolated on a logarithmic intensity scale (Figure S11) to accurately infer the band edge position for perovskites.^46,47^ We plotted the WF and VB onset of our samples in Figure 2b with the energy band diagram illustrated in Figure 2c,d. The conduction band minimum (CBM) was positioned given the optical band gap that was obtained from the Tauc plot for control and TOPO-treated samples given in Figure S2b. The values of the WF, VBM (valence band maximum), and CBM are summarized in Table S4. We observed a decrease in WF in both OAI and TOPO-treated samples. This is further confirmed by Kelvin probe measurement (Figure S12 and Table S5).

Moreover, as discussed above, OAI treatment introduced a downward band bending of approximately 200 meV at the surface, resulting in a more n-type perovskite film, which is in line with a recent report.^48^ The addition of a TOPO layer, on the other hand, reversed the surface band bending into an upward one by around 100 meV. With the adjacent hole transport material (HTM), i.e. spiro-OMeTAD in this work, we observed that the ground state interfacial energy levels exhibit dramatic changes in both samples, which is believed to be caused by charge carrier rearrangement at the interface of perovskite and HTM, as observed in our previous studies.^49,50^ It is also noted that the abrupt increase in vacuum level at the interface is almost identical for both samples by around 870 meV. In order to approach the interfacial energy level alignment under device operating conditions, we further conducted ultraviolet photoelectron spectroscopy (UPS) measurements under additional white light with light intensity equivalent to 1 sun (dashed line in Figure 2e). The energy levels of the spiro-OMeTAD layer are found to exhibit dramatic downward shifts by 0.66 and 0.62 eV for the control and TOPO-treated samples, respectively. Such shifts, as recently observed in perovskite/organic semiconductor interfaces,^49,50^ are caused by charge carrier accumulation at the interface under illumination, which leads to a realignment of the energy levels at perovskite/spiro-OMeTAD interface with spiro-OMeTAD HOMO shifting toward the perovskite VBM. It is noted that the measured energy levels from photoemission always refer to the Fermi level of the conductive FTO substrate. Given the unchanged perovskite energy levels upon white light illumination, the large energy offset between perovskite VBM and spiro-OMeTAD HOMO is then significantly reduced to 210 meV for the control and to 260 meV for TOPO-treated samples.

So far, we have revealed two roles played by the TOPO layer. One is its function as a dipole pointing toward the perovskite film, leading to a decrease in WF compared to the control sample, confirmed from both UPS (∼130 meV) and Kelvin probe measurements (∼210 meV). The influence of tuned WF on device stability was studied in a recent paper.^48^ It was reported that a lower WF, i.e. a less negative vacuum level, reduces the halide migration activation energy and thus leads to more pronounced hysteresis and less device stability.^48^ This is not what we observed in our work. We detected a decrease in the WF for TOPO-treated samples. The other role of TOPO is the chemical bonding with surface ions in perovskite that leads to an upward surface band bending of 100 meV. This change in interfacial energy level alignment is of great interest. In the following, we discuss its effect on charge exaction and charge selectivity and, most importantly, on device stability.

Charge carrier selectivity at the interface of our samples was characterized by using transient surface photovoltage spectroscopy (tr-SPV). The tr-SPV signals are directly proportional to the separated charges (SPV(*t*) ≈ (*n*(*t*) – *p*(*t*)) × 0.5*L*) at the buried interface of a device (*L* is the thickness of the light absorber layer, i.e. the perovskite film in our study). Therefore, transient SPV provides important insights into the dynamics of charge extraction and recombination.^51,52^ This technique was employed in our previous publication on the comparison of a range of self-assembled monolayers (SAM) comprising hole transport materials for the efficiency of hole extraction.^37^ We measured transient SPV for several different samples, i.e. control perovskite films, TOPO-treated perovskite films, control perovskite films with spiro-OMeTAD, and TOPO-treated perovskite films with spiro-OMeTAD (all deposited on glass/TiO_2_). We also conducted the measurement for samples deposited on glass (Figure S13). It shows that CsPbI_3_ films deposited on glass have shallow and deep trap states^53−55^ (with activation energy up to 0.8 eV) while the TiO_2_ substrate helps to remove these trap states and results in clean and sharp SPV contour plots at a wide spectral range from 1.8 to 3.0 eV.

Figure 3a–c exhibits the penetration depth of three different photon energies used in tr-SPV characterization. The penetration depth is calculated from the absorption coefficient of perovskite films (Figure S2d) as the top HTM has an absorption onset of approximately 2.92 eV.^56^ It shows that at red light (1.8 eV) excitation it can penetrate close to the middle of the film. At green light (2.2 eV) excitation, it penetrates approximately 74 nm deep into the film. At blue light (2.6 eV), it penetrates within 10 nm next to the interface. Figure 3d–f presents the tr-SPV results of samples measured under these three photon energies at fluences of 0.010, 0.02, and 0.040 μJ/cm^2^, respectively, corresponding to the carrier concentration of 1.4 × 10^15^ cm^–3^ close to 1 sun operation conditions. Such a measurement was also conducted at 0.1 sun and 10 suns equivalent (Figure S14), as discussed in the SI. Thus, the larger photon energies (i.e., 2.2 and 2.6 eV) generated charge carriers closer to the HTM. This includes free carrier nonuniformities in the perovskite film, demands a longer charge diffusion distance, and results in lower SPV amplitude in the same samples. However, we observed the same trend for the measurement conducted at three photon energies and three light intensities, where TOPO-treated samples have higher SPV amplitude than the control samples.

**Figure 3:**
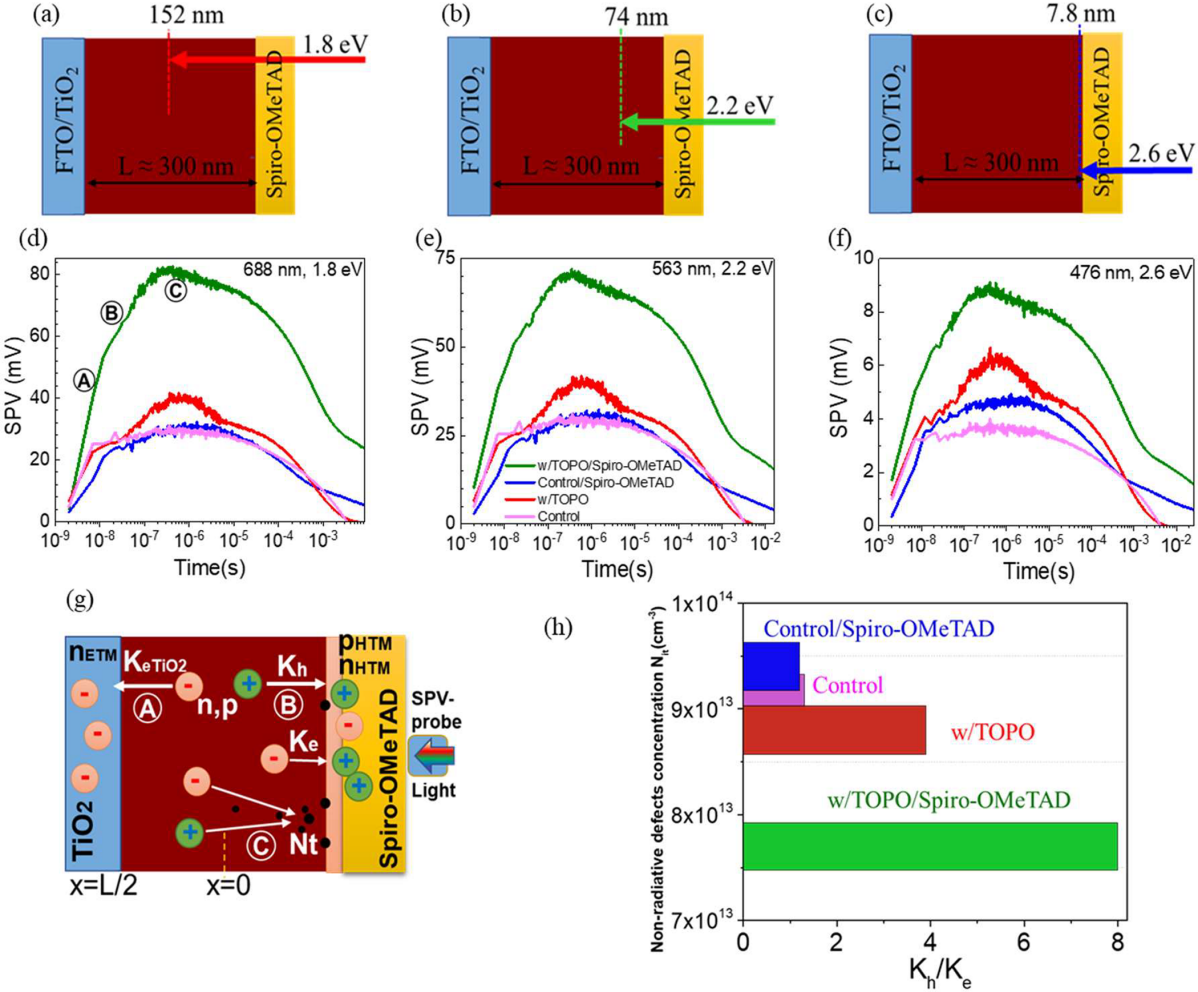
Charge selectivity. Scheme of light penetration depth into the samples for (a) red light (688 nm, 1.8 eV), (b) green light (563 nm, 2.2 eV), and (c) blue light (476 nm, 2.6 eV). Transient SPV of control (pink line), w/TOPO (red line), control/spiro-OMeTAD (blue line), and w/TOPO/spiro-OMeTAD (green line) measured at excitation sources of (d) 688 nm (1.8 eV), (e) 563 nm (2.2 eV), and (f) 476 nm (2.6 eV) at fluences of 0.010, 0.029, and 0.040 μJ/cm^2^, respectively, equivalent to 1 sun light intensity. (g) Charge extraction and recombination model for transient SPV data fitting describing carrier transport to ETL and HTM layers at two electrodes. The rate constant *K*_eTiO2_ characterizes electron injection from perovskite to the TiO_2_ layer (process A). The constants *K*_e_ and *K*_h_ correspond to electron and hole injection rates from the perovskite to the HTM side (process B). Defect concentration *N*_t_ is responsible for SRH nonradiative recombination (process C). The extracted electron and hole concentrations *n*_etm_ and *p*_htm_ induce the simulated tr-SPV signal as shown in Figure S15 by black curves (more details are given in eq S7). (h) Ratio of *K*_h_ to *K*_e_ and its correlation to nonradiative defect concentration, extracted from the fitting.

In general, the SPV signal for efficient HTM (or ETM) appears as a fast exponential rise with a large amplitude. The recombination and nonefficient charge selectivity tend to slow down the rise and decrease the SPV amplitude. We observe a significant boost of hole extraction in the TOPO-treated sample in the presence of adjacent HTM, approximately 2 times higher in the SPV amplitude and much faster signal rise than that of control samples at all studied laser photon energies. In contrast, for control samples with and without spiro-OMeTAD HTM, the SPV amplitude is almost the same. The rise in the SPV amplitude in the control sample with HTM starts to be noticed under blue light illumination. In other words, for control samples, holes can be properly extracted only in the vicinity of HTM. Furthermore, TOPO-treated samples even without HTM showed a higher SPV amplitude than the control and with HTM. This behavior of TOPO-treated devices implies the positive effects of TOPO treatment on free electron extraction, hole extraction, and charge recombination.

To explain in detail charge extraction dynamics, we developed a 1D simulation of charge. We will mainly focus here on the processes that occurred during the 1 ns to 10 μs time scale (4 orders of magnitude) associated with carrier extraction and recombination. The initial charges are generated in the perovskite layer by the light and can be extracted to ETM and HTM with extraction rate constants *K*_eETM_ and *K*_h_, respectively, as shown in Figure 3g. To describe the charge selectivity properties at HTM we also introduced the electron injection rate constant *K*_e_ responsible for electron injection to HTM (for example, as a result of poor selectivity). The nonradiative and radiative charge recombinations are characterized by defect concentration *N*_T_ and band-to-band recombination *C*_b_. To simplify the fitting procedure, we adopted part of the constants from the literature^55,57^ (see Table S6). More details about the model can be found in the SI. The results of the fit and main fitting parameters are summarized in Figure S15 (black curves) and Table S7.

According to the model results, the fast initial rise (region A in Figure S15 is formed due to rapid electron extraction to TiO_2_ with a rate constant (*K*_eETM_) of 1.8 × 10^7^ s^–1^. The electron extraction rate constant only slightly decreased to 1.3 × 10^7^ s^–1^ in other devices, signaling that other processes are responsible for dramatic carried dynamics changes in time regions B and C. The difference in *K*_eETM_ can originate from a slightly faster electron extraction at the interface of TiO_2_ and CsPbI_3_ due to enhanced energy offset at the CBM level as characterized by the UPS measurement.^56^

We further found that TOPO treatment significantly boosts the free holes extraction rate (*K*_h_) and selectivity properties of HTM. The value of *K*_h_ increased nearly twice in TOPO-treated samples with spiro-OMeTAD (8 × 10^6^ s^–1^) compared with the control sample (4.4 × 10^6^ s^–1^). The improved hole extraction near the TOPO surface is also observed in TOPO-treated perovskite films (3.9 × 10^6^ s^–1^) in comparison with the control sample (1.5 × 10^6^ s^–1^). In addition to improved hole extraction, TOPO significantly boosts the selectivity properties of HTL, which can be characterized by the *K*_h_/*K*_e_ ratio where *K*_e_ is electron injection in the HTM (Table S7). Figure 3h shows that TOPO treatment increased the *K*_h_/*K*_e_ ratio from a value of 1.3 in the control to a value of 3.9 for perovskite films. Further, this value increased to 8 in the presence of spiro-OMeTAD; meanwhile, control samples without TOPO treatment had a rather low value of 1.2. Finally, we observe slight passivation of nonradiative recombination in TOPO-treated samples with spiro-OMeTAD (nonradiative defect concentration of 7.7 × 10^13^ cm^–3^) in comparison with control samples with spiro-OMeTAD (9.4 × 10^13^ cm^–3^). This is in good alignment with what we observed in the PL measurement, as presented in Figure 1a.

The overall picture of the charge dynamics suggested by tr-SPV with simulation demonstrates a significant role for TOPO in the improvement of hole extraction. The TOPO layer stimulates free hole extraction in the HTM and repels electrons from the TOPO surface. The selectivity of neat HTM can result in noticeable suppression of charge recombination near the HTM surface and in the HTM itself. TOPO also provides chemical passivation of traps (18% decrease in the trap concentration). We believe that the suppressed recombination is mainly contributed by the enhanced hole selectivity due to the formation of upward surface band bending at the interface between CsPbI_3_ and spiro-OMeTAD. The enhanced hole selectivity originates from better energetic alignment caused by upward surface band bending and dipole activity which contributes to recombination suppression as well.

Figure 4a shows the scheme of the device structure, with a compact TiO_2_ layer as the electron transport material (ETM) and spiro-OMeTAD as the hole transport material (HTM). The TOPO molecule is represented on top of the perovskite film with the alkyl chains visible. All the samples, with and without the TOPO, are treated with OAI to passivate the defects of the perovskite, as reported previously.^30^ The champion device *J–V* curves in Figure 4b show a clear improvement of *V*_oc_ with TOPO treatment. Figure S16b shows the EQE spectra of the champion devices with their *J–V* curves given in Figure S16a. The improved hole extraction is found to significantly improve the *V*_oc_ and the FF of the cells as presented in the box chart and distribution curves in Figures S17 and S18a–c and Table S8. Moreover, we achieved a *V*_oc_ of over 1.2 V under the TOPO treatment, while control samples showed a *V*_oc_ strictly below 1.2 V, similar to what was reported in refs (39 and 58); *J–V* curves with *V*_oc_ values of over 1.2 V are given in Figure S19.

**Figure 4:**
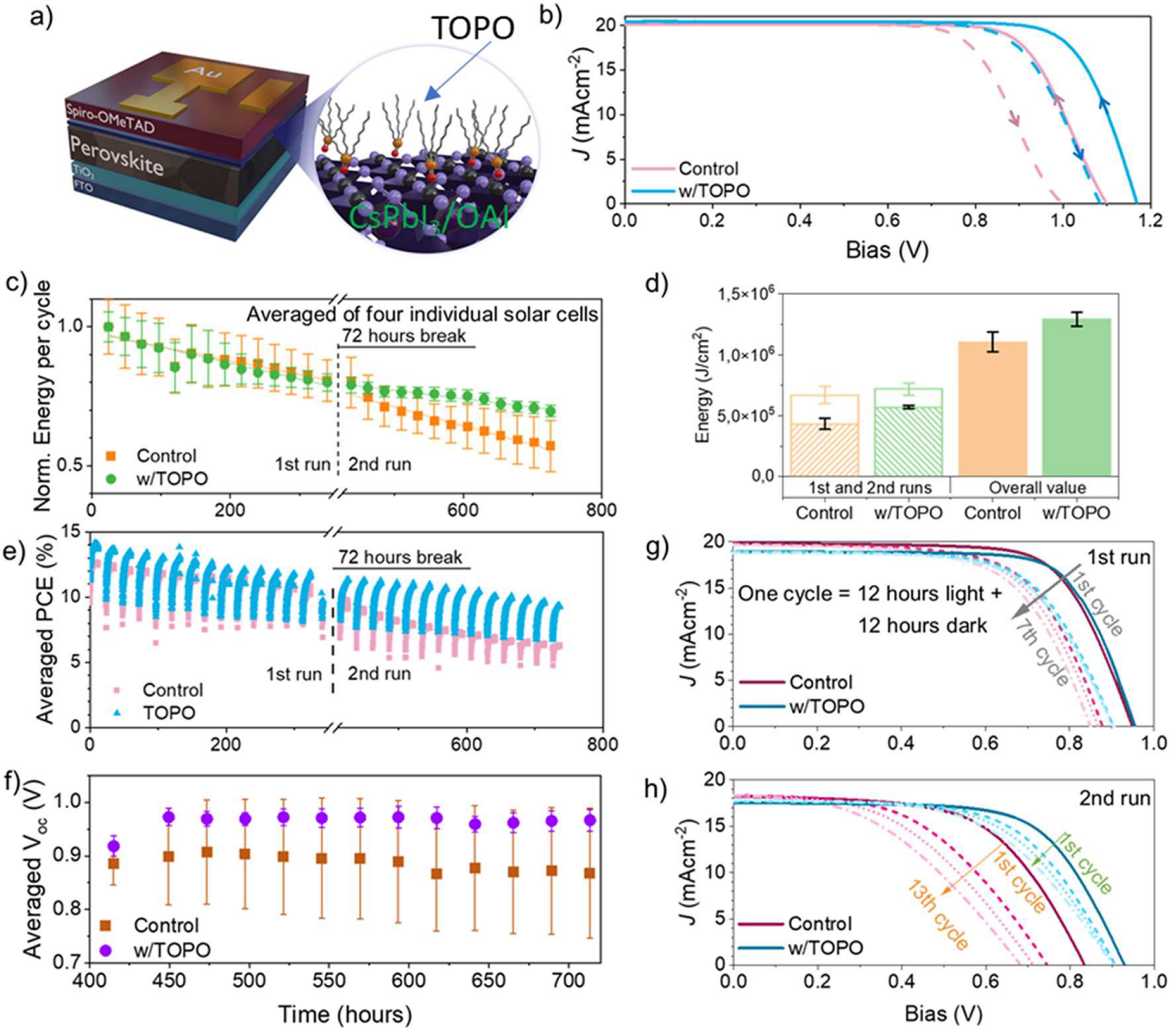
*J–V* measurements and device stability. (a) Scheme of the device structure with the highlighted part for the TOPO treatment on the perovskite surface. (b) The champion *J–V* curve (100 mV/cm^2^, AM1.5G, scan rate 200 mV/s, reverse scan and forward scan indicated as solid and dashed lines, respectively, and further indicated in arrows), The device performance values for the champion device are given in Figure S23. (c) Normalized energy output per cycle, with each cycle composed of 12 h light-on and 12 h light-off, (d) energy output in the first and second runs and the overall value, (e) averaged efficiency, and (f) evolution of averaged *V*_oc_ as a function of aging hours in the second run. (c–f) are averaged from four individual solar cells. *J–V* curve evolution as a function of illumination cycles in (g) the first run and (h) the second run for the control and TOPO-treated samples. The dashed lines in (g) and (h) refer to the number of illumination cycles, with the last cycle at 13 for both samples.

We focused on the stability of CsPbI_3_, which is more critical than the organic–inorganic perovskite compositions.^31,59^ The statistical analysis of the device performance parameters is reported in Figure S21a–c in the SI, and it is consistent with the performance of the champion devices. Device stability was measured in a custom-built MPPT (maximum power point tracking) aging system^60^ according to ISOS-LC-1l,^6^ by alternating 12 h of illumination under 1 sun and 12 h of dark for 27 cycles separated in two runs with a break of 72 h. We conducted the stability test under nitrogen and at an ambient temperature of 25 °C including the 72 h break in the dark phase for all measured samples. More details of the long-term stability test can be found in the SI. Moreover, constant light illumination MPPT was also measured as shown in Figure S22 The UV component of the solar spectrum (Figure S24) was filtered to prevent degradation caused by TiO_2_ oxygen desorption.^61,62^Figure 4c shows the energy produced per cycle normalized to the first cycle. Each point is an average of the energy produced by four independent devices (the individual data of each sample and the applied method to filter data, as well as a full statistic evaluation, are given in the SI and Figure S20a,b). In the first run (i.e., 14 cycles in 342 h), we observe a similar degradation trend for both samples, while a much slower degradation process is observed in TOPO-treated samples in the second run (i.e., 15th–27th cycles in 322 h). The overall energy generated by the solar cells is averaged and is plotted in Figure 4d. Overall, TOPO-treated solar cells can generate 16.8% more energy than the control, with the surplus energy mainly generated after the 342 h (33.3% over the control). It should be noted that control samples have a larger deviation than TOPO-treated ones. We took one representative sample from each group and plotted the PCE normalized to the initial value as shown in Figure 4e. The evolution of the PCE follows the same trend as the produced energy in Figure 4c.

We observed robust *V*_oc_ and FF of TOPO-treated solar cells in the long-term stability test as well, as presented in Figure 4f and Figure S20d. It shows that a significantly more stable *V*_oc_ is observed in TOPO-treated samples compared to the control since the 414th hour of the stability measurement. Figure 4g,h presents the *J–V* curve evolution of control and TOPO-treated samples during the first and second runs, respectively. We can see that both samples exhibit good stability in the initial cycles. Yet the superior stability in TOPO-treated samples becomes more pronounced from the 15th cycle. In the 14 overall cycles in the second run, the control sample degrades significantly, with a drop mainly in *V*_oc_. In contrast, TOPO-treated samples exhibit only a slight decrease in *V*_oc_. The stability of the control in the first 342 h is consistent with the literature that used OAI to passivate the defects of the perovskite. Nevertheless, on a longer time scale, the CsPbI_3_ passivation solo is not sufficient to guarantee stability. The reason behind the improved stability of the TOPO sample will be investigated in the following paragraph.

So far, we have demonstrated that a better charge selectivity leads to better device stability for TOPO-treated samples. The reasoning behind this can be understood in the following. We noted a much more severe *V*_oc_ loss (Figure 4h) and FF loss (Figure S20d) in the *J–V* curves of control samples, with respect to the TOPO samples, recorded from the first to the 13th cycle during the aging test. In particular, the FF loss is mainly coming from the increase in serial resistance as the slope of the *J–V* curve at the bias around the *V*_oc_ becomes less and less steep over the cycled illumination. The increase in serial resistance implies less conductivity in contact layers such as the ETM, HTM, or metal electrode. We suggest that it is caused by iodide migration across the interface to the HTM layer during illumination and under an applied bias.^63^ It was reported that the chemical reaction happens between spiro-OMeTAD^+^ and migrating I^–^, which progressively reduces the conductivity in HTM.^63,64^ With the TOPO treatment, the resulting upward band bending due to the chemical bonding increases the energy barriers for electrons and negatively charged mobile ions, such as iodide, to move across the interface. This prevents film conductivity drops in HTM and thus holds the initial FF robustly in TOPO samples. Besides, the iodide diffusion into spiro-OMeTAD will shift up the HOMO levels toward the vacuum level because the radical concentration of spiro-OMeTAD^+^ will be reduced due to the reaction with iodide and that leads to less p-doping in spiro-OMeTAD. Then the interfacial energetic level realignment will create more nonradiative recombination, which leads to severe *V*_oc_ loss.^34^

Additionally, by chemical bonding with iodine vacancies at the interface, TOPO helps to make iodine vacancies less mobile. Iodine vacancies at the interface are found to be benign, but illumination can promote the diffusion of iodine vacancies to the bulk, making them detrimental for creating new nonradiative recombination centers.^65^ This causes severe loss of *V*_oc_ in control samples but is effectively suppressed in TOPO samples. Third, the energetic upward band bending enhances the charge selectivity, i.e. better separation between electrons and holes at the interface, which leads to less charge accumulation near the interface and thus less interfacial recombination, as supported by tr-SPV data in Figure 3. In return, it contributes to better stability in devices.^66^ The synergy of the above effects of TOPO provides better device stability and performance in the TOPO-treated samples.

Herein, we developed a strategy based on surface-dipole-induced band bending to mitigate the charge loss at the perovskite/HTL interface. We have demonstrated that the TOPO molecule causes surface upward band bending and resolves unfavorable downward bending induced by OAI passivation of the CsPbI_3_ film. The TOPO dipole molecule shifts the interfacial energy levels and reduces the energy offset between the perovskite VBM levels and spiro-OMeTAD HOMO levels shown by UPS measurements. Further, this energy level alignment induces a boost in charge transfer at the interface, as revealed by the trSPV study. The competing effect of passivation and charge extraction is resolved by charge transport simulation, proving that TOPO plays a minor role in trap passivation and mainly significantly boosts the hole extraction rate. By applying the TOPO dipole at the HTL interface in all-inorganic perovskite solar cells, we have achieved a *V*_oc_ of over 1.2 V with improved stability, 19% efficiency, and 16% more energy production for the TOPO-treated samples. This work reveals that besides defect passivation interfacial energy level alignment and charge selectivity play a critical role in device stability and efficiency.

## Supporting Information Available

Detailed experimental method and device fabrication method, summary of a literature survey, thin film characterization, PL and trPL measurements with ETL/HTL, XPS analysis, Kelvin probe measurements, contour plots of the transient SPV illuminated by different light energies, transient SPV at 0.1 sun and 10 suns, transient SPV fitting results, cChampion *J–V* of CsPbI_3_ solar cells and EQE, box charts of PCE, *J*_sc_, *V*_oc_, and FF, long-term stability data-I “best PCE+stability filter”, long-term stability data-II “PCE distribution filter”, constant illumination MPP, main fitting constants of simulation, simulation of charge extraction and recombination, penetration depth for perovskite thin film, and QFLS calculation (PDF)
