# Supplementary material for: Interface Modification for Energy Level Alignment and Charge Extraction in CsPbI3 Perovskite Solar Cells
Source: ACS Energy Lett. 2023 Sep 22;8(10):4304–14. doi: 10.1021/acsenergylett.3c01522 (PMC10580311; doi:10.1021/acsenergylett.3c01522)
Supplement: Supplementary file 1 — nz3c01522_si_001.pdf [file nz3c01522_si_001.pdf]

Supporting Information

Interface Modification for Energy Levels Alignment and Charge Extraction in $CsPbI_3$ Perovskite Solar Cells

Zafar Iqbal[1], Fengshuo Zu[2], Artem Musiienko[1], Emilio Gutierrez-Partida[3], Hans Köbler[1], Thomas W. Gries[1], Gennaro V. Sannino[1,6], Laura Canil[1], Norbert Koch[1,2], Martin Stolterfoht[3,4], Dieter Neher[3], Michele Pavone[5], Ana Belen Muñoz-García[6], Antonio Abate[1,7,8*], and Qiong Wang[1*]

[1] Helmholtz-Zentrum Berlin für Materialien und Energie GmbH. Hahn-Meitner-Platz 1, 14109 Berlin, Germany.

[2]Institut für Physik & IRIS Adlershof, Humboldt-Universität zu Berlin, 12489 Berlin, Germany.

[3]Institute for Physics and Astronomy, University of Potsdam, Karl-Liebknecht-Straße 24–25,

14476 Potsdam-Golm, Germany.

[4]The Chinese University of Hong Kong, Electronic Engineering Department, Shatin N.T., Hong Kong

[5]Department of Chemical Sciences, University of Naples Federico II, Comp. Univ. Monte S. Angelo, Via Cintia 26, 80126 Naples, Italy

[6]Department of Physics "Ettore Pancini", University of Naples Federico II, Comp. Univ. Monte S. Angelo, via Cintia 26, 80126 Naples, Italy

[7]Department of Chemistry Bielefeld University, Universitätsstraße 25, 33615 Bielefeld, Germany

[8]Department of Chemical Materials and Production Engineering, University of Naples Federico II, Piazzale Vincenzo Tecchio 80, 80125 Naples, Italy

Experimental section

Chemicals

CsI (99.999%, ABer), lead(II) iodide (PbI_2, 99.99%, TCI), dimethyl ammonium iodide (DMAI) (98%, Sigma-Aldrich), methyl ammonium chloride (MACl, Dyenamo), *n*-octylammonium iodide (OAI, GreatCell Solar), dimethylformamide (DMF, 99.8%, Sigma-Aldrich), dimethyl sulfoxide (DMSO, 99.9%, Sigma-Aldrich), isopropanol (IPA, 99.5%, Sigma-Aldrich), toluene (99.8%, Sigma-Aldrich), chlorobenzene (99.8%, Sigma-Aldrich), tris(2-(1H-pyrazol-1-yl)-4-tert-butylpyridine)cobalt(III) tri[bis-(trifluoromethane) sulfonimide] (FK209, Dyenamo), bis(trifluoromethane)sulfonimide lithium salt (Li-TFSI, Sigma-Aldrich), ethanol (99.9%, Merck), spiro-OMeTAD (Lumtec), titanium di-isopropoxide bis(acetylacetonate) (TIAP, 75 wt % in isopropanol, Sigma-Aldrich), 4-test-butyl pyridine (tBP) (98%, Sigma-Aldrich), tri-octylphosphine oxide (TOPO) (99%, Sigma-Aldrich).

All chemicals are used as received.

FTO substrates cleaning

Patterned FTO substrates (TEC 15, Yingkou company, with dimensions 2.5 cm × 2.5 cm) were numbered on the back side (glass side). These numbers were designated to every device (*e.g.* Z1, Z2, Z3, *etc.*). Substrates were cleaned with 2% Mucosal solution with a very fine brush to clean the FTO surface, then washed with distilled water to remove soap contents. Afterwards,

cleaned with acetone and isopropanol for 15 min by sonication. After drying with a nitrogen gun, the substrates were placed in a UV-ozone cleaner for 15 minutes right before the titanium oxide layer deposition.

Solution preparation

- 2% Mucasol solution was made by mixing 20 mL mucosal in 1000 ml distilled water.
- TiO_2 solution was prepared by adding 150 µL TIAP into 15 mL of ethanol.
- 1.0 M PbI_2 solution was prepared by dissolving 1.17 g of PbI_2 solution in 2.422 ml DMF solvent. The mixture was stirred at 80 °C for 2 hours to get the PbI_2 solution.
- To get 0.60 M $CsPbI_3$ solution, 1.987 ml PbI_2 solution was added in 0.4370 g CsI salt and stirred for 10 min until it completely dissolved.
- To make 1:1:1 (atomic ratio) CsI: PbI_2: DMAI solution, 2.542 ml above $CsPbI_3$ solution was added in DMAI and stirred for 10 min until a clear, yellowish perovskite solution was formed.
- 45 mM MACl solution was made by dissolving 57.7 mg MACl salt in 19 mL IPA and was stirred for two hours until it completely dissolved.
- OAI solution was prepared by dissolving 3 mg of OAI in 1mL IPA and stirring for 1 hour.
- 20 mM TOPO solution was made by dissolving 15 mg TOPO in 1.940 ml toluene and other concentrations were made by using the dilution formula that is $M_1V_1 = M_2V_2$.
- A 36 mM solution of spiro-OMeTAD was prepared by dissolving 200 mg spiro-OMeTAD in 2.2 ml chlorobenzene with 87.78 µL tBP, 51.11 µl LiTFSI with a stock solution of 520 mg/ml in acetonitrile, and 22.22 µl FK209 with a stock solution of 375 mg/ml in acetonitrile.

Device Fabrication

TiO_2 compact layer

TiO_2 compact layer was deposited by spray pyrolysis with oxygen as the carrier gas. 16 substrates were placed on a hot plate fitted inside a fume hood. One edge of each substrate is covered by around 5 mm using a cover glass to keep the conductive FTO side exposed for low contact resistance. Then the substrates were heated up to 450 °C and were kept at this temperature for 15 min before and 30 min after the spray of the precursor solution. The whole solution was transferred into a spray nozzle and sprayed at roughly 20 cm away from the substrates with an inclination angle of 45 degrees, with at least 20 seconds of delay between each spraying cycle. Afterward, substrates were left to cool down to room temperature and then put in an ozone chamber for 15 minutes before perovskite film deposition.

Deposition of control perovskite films

After the ozone treatment, substrates were transferred into a glove box filled with nitrogen (O_2 <0.1 ppm, H_2O <0.1 ppm). The substrates were placed on a hot plate at 70 °C for 5 min before perovskite deposition. 80-100 µl perovskite solution was added and spin-coated quickly at 3000 rpm for 30 seconds. Then 350 µl MACl solution was dropped on the top and spin-coated for another 35 seconds. The wet films were then annealed in a dry air box with a relative

humidity (RH) of ~ 1% for 1 min at 210 °C. Afterwards, substrates were transferred back to a nitrogen-filled glove box, where 100 µl OAI solution was dropped on the top and spin coated at 5000 rpm for 30 seconds, followed by annealing at 100 °C for 5 min.

Perovskite films with TOPO treatment

After the control perovskite films cooled down to room temperature, 200 µl TOPO solution at varied concentrations was deposited by spin coating at 5000 rpm for 30 seconds. No annealing is needed for this step.

Hole Transport Layer (HTM) deposition

100 µl spiro-OMeTAD solution was deposited by spin coating at 3500 rpm for 30 seconds. No annealing is needed for this step.

Afterward, all the samples were transferred into a dry air box (RH ~ 0.1%) for oxygen soaking.

Deposition of metal contact

Gold was evaporated using a thermal evaporator under a vacuum of approximately $1*10^{-6}$ pa. The deposition rate was programmed at 0.02 Å/s for the first 1 nm, 0.1-0.2 Å/s for the following 5 nm, and then 0.5 Å/s until 20 nm and then 1Å/s for the rest of the deposition. Overall, it takes around 25 min for the deposition of 100 nm of gold. The active area of the device was 0.18 cm^2 defined by the shallow mask.

Solar cell characterization

The light source was provided by an Oriel LCS-100 class ABB solar simulator (1Sun, AM1.5G, 100 $mWcm^{-2}$) installed inside a nitrogen-filled glovebox. Before the light *J-V* measurement, the light intensity was calibrated with a silicon reference cell (Fraunhofer ISE). A Keithley power meter (2400 SMU) was used for the bias application to solar cells for the *J-V* scans, programmed by LabView. The bias was applied to scan from 1.25 V to -0.1 V back and forth, with a scan rate of 200 mV/s and a step size of 0.02 V.

EQE measurements

EQE spectra were recorded with the TracQ-Basic software, connected to the light source (Oriel Instruments QEPVSI-b system integrated with a Newport 300 W xenon arc lamp) with an optical fiber. The spectrum of the light source was calibrated with a Si reference cell with a known spectral response before the measurement. The monochromatic light was provided by a Newport Cornerstone 260 monochromator with a chopping frequency of 78 Hz.

Absolute Photoluminescence Spectroscopy (PL)

A 445 nm CW laser (Insaneware) was used as the excitation source for the PL measurements with an optical fiber connected to an integrating sphere where samples were loaded. Samples for the PL measurements were encapsulated with a cover glass before being taken out of a nitrogen-filled glovebox.

Time-resolved photoluminescence spectroscopy (TRPL)

TRPL signals were acquired with a TCSPC system (Berger & Lahr) after excitation with a mode-locked Ti: sapphire oscillator (Coherent Chameleon) that provides a pulse-picked and frequency-doubled output, with nominal pulse durations ~ 100 fs and fluence of ~ 30 nJ/cm^2 at a wavelength of 470 nm.

X-ray and Ultraviolet photoelectron spectroscopy (XPS and UPS)

Ultraviolet photoelectron spectroscopy (UPS) was conducted using a monochromated helium discharge lamp (HIS 13 FOCUS GmbH, photon energy of 21.22 eV) in an ultrahigh vacuum system (base pressure of 1 × 10^{-9} mbar). With a monochromator, the visible light was eliminated and UV flux was significantly reduced (attenuation by a factor of ca. 100 folds as compared to that of the standard helium lamp). X-ray photoelectron spectroscopy (XPS) was performed using a standard Mg Kα radiation (1253.6 eV, anode power of 20 W) generated from a twin anode X-ray source. All spectra were recorded at room temperature and normal emission using a hemispherical electron analyzer (SPECSPhoibos 100). The illumination experiments were conducted using a white halogen lamp (Solux MR16 4700K, 50 W, daylight rendering) during UPS measurements with an intensity of ca. 100 mW/cm^2. The secondary electrons cutoff (SECO) spectra were conducted at a negative bias of 10 V.

Kelvin probe measurement for Work Function

The measurement of WF was performed by the non-contact and non-destructive Kelvin probe method, in which the sample and probe form a parallel plate capacitor.[1]

Kelvin Probe Force Microscopy (KPFM)

KPFM was recorded in an nitrogen-filled glovebox on a Bruker MultiMode mircroscope. Pt-Ir-coated cantilever tips (Bruker SCM-PIT,f_0 = 75 kHz, k = 2.8 N/m) with a tip radius of 25 nm were calibrated with respect to freshly cleaved HOPG. Determination of the CPD values succeeded via Gaussian fitting of the histograms extracted from the (2 x 2) μm^2 images.

Transient surface photovoltage (tr-SPV) measurements

Charge extraction in the time range of 5 ns up to 0.5 s was studied by non-contact SPV measurements exited by 5 ns above bandgap laser (1.8 eV). We used fluences of 0.072 μJ, which corresponds to a carrier concentration of 3×10^{15} cm^{-3} close to 1 sun operation conditions. Detailed SPV setup description is given in our previous study [1,2]. Contour plots were recorded with a tunable laser in the range 0.6-3 eV using fluences of 72 μJ to ensure a good signal-to-noise ratio.

Simulation of charge extraction and recombination

Eq. S1-6 describe the simulation model for charge separation, trapping, and recombination where n and p are the concentration of photo-induced electrons and holes. The constants K_e and K_h correspond to electron and hole injection rates from perovskite to HTM. The constant K_{eTiO} corresponds to electron injection rates from perovskite to ETM (TiO_2). Similarly, K_{eb} and K_{hb} are reinjection rates of electron and hole to perovskite from HTM, which effectively include back tunneling/thermionic emission, and diffusion of the free carriers, as well as the

drift of the free carriers due to the presence of the space charge. $C_b$is radiative recombination constant. N_t and σ are concentrations and capture a cross-section of defects responsible for SRH non-radiative recombination. τ_{HTM} and τ_{ETM} characterizes the carriers' lifetime in HTM and ETM. The system of the equations cannot be solved analytically; therefore, we used the Adams backward differentiation formula (BDF) solving algorithm. We used the Levenberg-Marquardt method to fit constants with minimal deviation from experimental SPV results. SPV data were extrapolated logarithmically for better fitting results. The results of the fit are given in **Fig. S15** and summarized in **Table S7**.

$$\frac{dp}{dt} = -K_h p + K_{hb} p_{HTM} - C_b(pn) - p\sigma_{ht} v_h n_t \quad \textbf{(Eq. S1)}$$

$$\frac{dn}{dt} = -K_e n + K_{eb} n_{HTM} - C_b(np) - n\sigma_{et} v_e (N_t - n_t) - K_{eTiO} n \quad \textbf{(Eq. S2)}$$

$$\frac{dp_{HTM}}{dt} = K_h p - K_{hb} p_{HTM} - \frac{p_{HTM}}{\tau_{HTM}} \quad \textbf{(Eq. S3)}$$

$$\frac{dn_{HTM}}{dt} = K_e n - K_{eb} n_{HTM} - \frac{n_{HTM}}{\tau_{HTM}} \quad \textbf{(Eq. S4)}$$

$$\frac{dn_{ETM}}{dt} = K_{eTiO} n - \frac{n_{ETM}}{\tau_{ETM}} \quad \textbf{(Eq. S5)}$$

$$\frac{dn_t}{dt} = n\sigma_{et} v_e (N_t - n_t) - p\sigma_{ht} v_h n_t \quad \textbf{(Eq. S6)}$$

Due to $d_{PER} \gg d_{HTM}$, $d_{PER} \gg d_{ETM}$, and assuming a uniform distribution of charges in perovskite, ETM, and HTM layers; so SPV signal can be simplified in the form:

$$SPV = \frac{L\, n_{ETM}}{2\varepsilon_{TiO}\varepsilon_0} + \frac{Ln_{HTM} - dp_{HTM}}{2 \quad \varepsilon_{HTM}\varepsilon_0} + \frac{Ln - p - nt}{2 \quad \varepsilon_{pero}\varepsilon_0} \quad \textbf{(Eq. S7)}$$

Where $L = d_{PER}/2$ -charge separation distance.

Scanning electron microscope (SEM)

The SEM images were recorded with the Hitachi S-4100 at an acceleration voltage of 5 kV.

X-ray diffraction (XRD)

X-ray powder diffractometer Bruker D8 Advance in Bragg-Brentano geometry with Cu Kα as the target and LYNXEYE as the detector was used for the XRD measurement, at a voltage of 20 kV and current of 5 mA, The samples were scanned from 5° to 70° with a step size of 0.01°.

UV-vis spectroscopy

Perkin Elmer LAMBDA 1050 UV/VIS spectrometer was used for the transmittance measurements of samples. Samples were encapsulated with a cover glass before being taken out of a nitrogen-filled glovebox for the UV-Vis measurements.

Ageing of Solar Cells (long-term stability measurement)

Solar cells were aged in a custom-built High-throughput Ageing Setup.[3] A light-cycling experiment according to ISOS-LC-1I [4] was performed with cycles of 12 h illumination phase

followed by 12 h of dark phase. During the illumination phase, special electronics were used to MPP-track all cells individually. A perturb and observe algorithm [5] with a delay time of 1 s and a voltage step-width of 0.01 V was applied to track the MPP. PCE_{MPP} values were taken every 2 min for all cells automatically. During the dark phase, cells were fully shaded with an automatic shutter system and disconnected from the MPP trackers.
Additionally, *JV*-scans, with a scan speed of 90 mV/s, were performed on every cell after 11 hours of the light phase of a cycle. During the dark phase, the shutter was shortly opened to perform *JV*-scans on selected pixels after 11.5 hours of darkness.
Devices were always kept at 25 °C with the help of actively controlled Peltier elements. Solar cells' active areas were touching a heating pad for direct thermal coupling. Aging was performed under a continuous flow of nitrogen in a closed box, no additional encapsulation was used. Sunlight with 1 sun intensity was provided by a metal-halide lamp using a H6 filter. A UV-blocking foil was used to block UV light with wavelengths below 380 nm. **Fig. S24** shows the spectrum of the light source in comparison to AM1.5G. The light intensity was actively controlled with the help of a silicon irradiation-sensor which was calibrated using a KG3 silicon reference cell from Fraunhofer ISE.

Supplementary data

Dipole formation at the interface between TOPO and perovskite

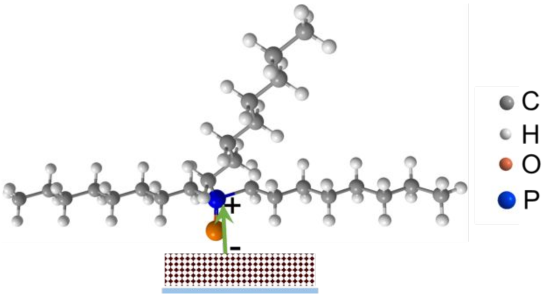

Scheme S1. Representation of TOPO and the relative dipole formed at the interface with perovskite. The dipole is defined as pointing to the positive side.

Summary of the Literature review (Table S1): The molecules OAI and TOPO are being used as passivating layers for the perovskite surface separately. In our work, we have used OAI and TOPO together in our champion device.

S.No	Perovskite composition	OAI/ TOPO	Function	PCE (%)	Year	Ref.
1	$(Cs_{0.05}(MA_{0.17}FA_{0.83})_{0.95}Pb(I_{0.83}Br_{0.17})_3)$	OAI	Passivating layer	15.1	2017	6
2	$(FAPbI_3)_{0.85}(MAPbBr_3)_{0.15}$	OAI	Stress Relaxation	21.5	2019	7
3	[(FAPbI3)0.95(MAPbBr3)0.05]	OAI	Post-treatment	22.0	2019	8
4	$FA_{0.65}MA_{0.35}PbI_{3-\delta}Cl_{\delta}$	OAI	Passivating layer	20.9	2021	9
5	$CsPbI_3$	OAI	Surface passivation	20.3	2021	10
6	$MAPbI_3$	TOPO	Post-healing of defects	12.8	2018	11
7	$MAPbI_2Br$	TOPO	Halide segregation	-	2018	12

8	$FA_{0.83}MA_{0.17}Pb_{1.1}I_{2.86}Br_{0.34}$	TOPO	Passivating layer	18.4	2022	13
9	$Cs_{0.05}FA_{0.79}MA_{0.16}Pb(I_{0.83}Br_{0.17})_3$	TOPO	Passivating layer	18.3	2020	14
10	$MA_{0.75}FA_{0.25}PbI_3$ (single crystal)	TOPO	Surface-Passivating of Single-Crystal	15.6	2022	15
11	$CsPbI_3$	TOPO	Surface passivation	14.0	2019	16
12	Cs0.05[MA0.15FA0.85PbI0.85Br0.15] 0.95	TOPO	Surface passivation study	-	2020	17
13	$CsPbI_3$	OAI: TOPO	Interface design	19.0	2023	This work

Inorganic nature of $CsPbI_3$

We first confirmed the crystal phase structure of our bare perovskite films *via* XRD. As shown in **Fig. S1a**, the sample presents dominating diffraction peaks at 14.35° and 28.86°, which corresponded to the (100) and (220) facets of γ-$CsPbI_3$ (*Pbnm* space group) crystallites[18]. The two broad peaks at 9.73° and 21.57° are likely to be (100) and (111) facets of δ-$CsPbI_3$ (yellow phase) with amorphous features [18,19]. This could be caused by the slow penetration of moisture into the dome filled with nitrogen during the XRD data measurement. The diffraction peak of $DMAPbI_3$ at 2theta of around 12° as reported in reference [20,21] was not observed here, indicating that the DMA^+ cation mostly vanished after the annealing.

We then conducted XPS measurement to further examine if any trace amount of organic moieties were left from DMAI or MACl in the pristine $CsPbI_3$ perovskite. **Fig. S1b** shows no signals for N 1s peak at the surface (< 10 nm) of $CsPbI_3$ thin films.

Together with the XRD data that revealed the absence of $DMAPbI_3$ in the bulk of $CsPbI_3$ thin films, we proved the chemical nature of $CsPbI_3$ inorganic perovskite.

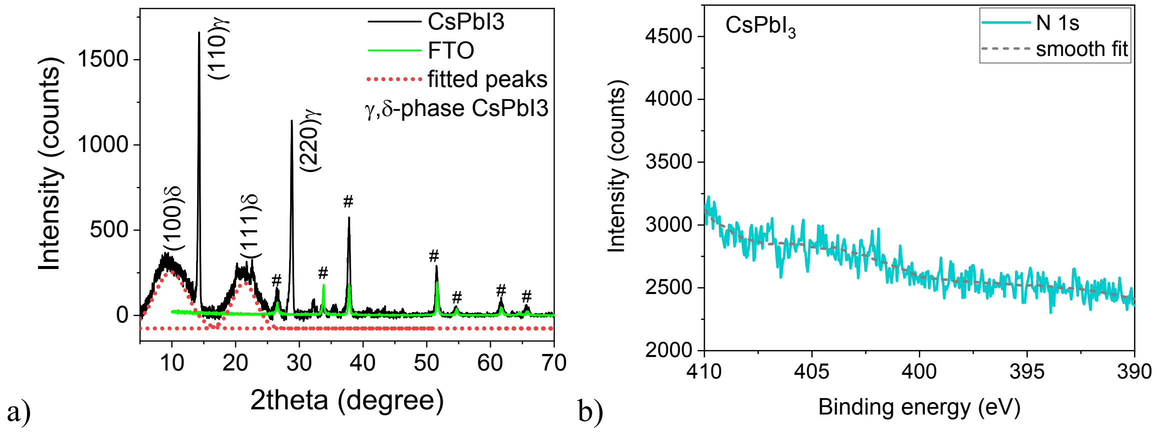

Fig. S1. **a)** XRD spectra of $CsPbI_3$ film deposited on TiO_2-covered FTO. **b)**. XPS of N 1s core-shell spectrum of as-obtained $CsPbI_3$ sample after annealing in a dry air box.

Optical bandgap of $CsPbI_3$

An optical bandgap of around 1.70 eV is observed from the Tauc plot and the EQE spectra, which is a little higher than the reported bandgap for γ-$CsPbI_3$, *i.e.* 1.69 eV. This is likely due

to slow degradation in samples during the storage and transportation as reported in reference [18].

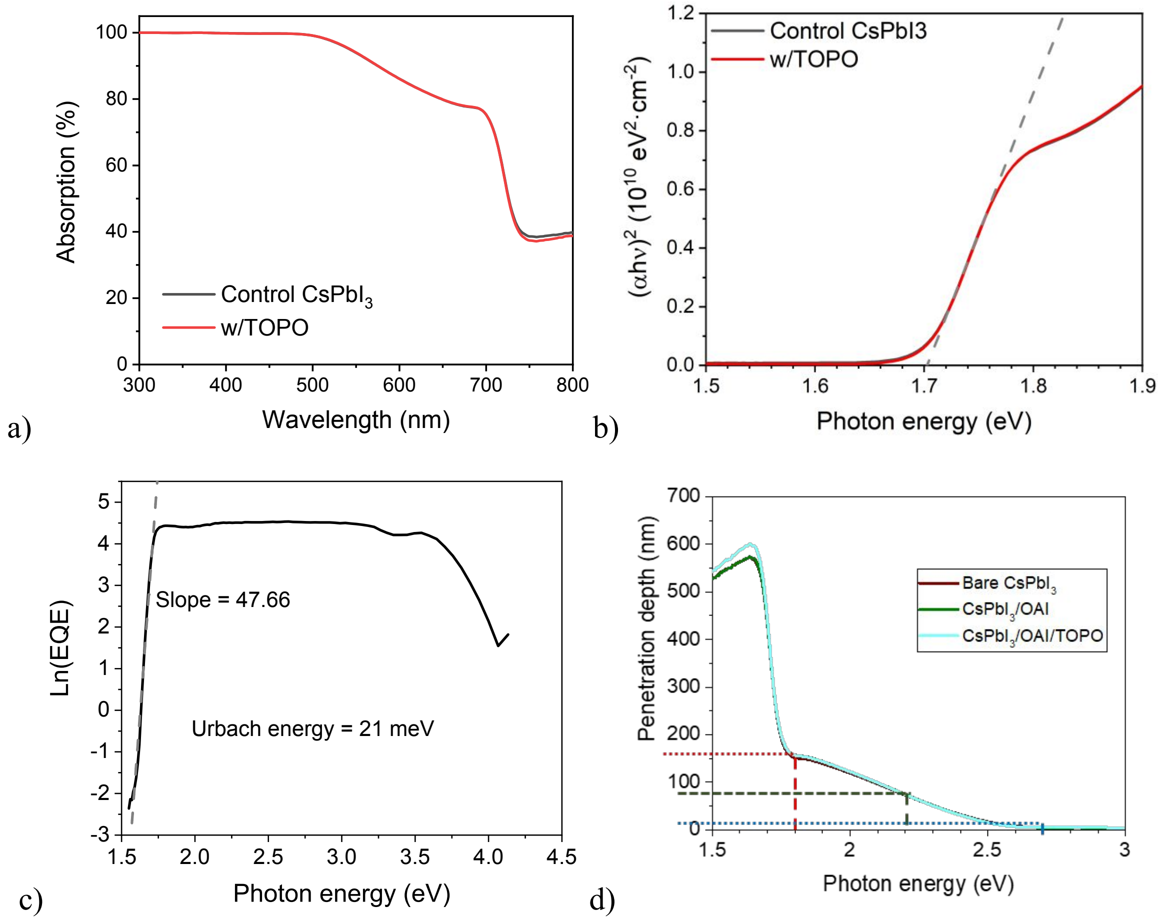

Fig. S2. a) Absorption spectra and b) Tauc plot of control and TOPO-treated $CsPbI_3$ perovskite films. c) Urbach energy of $CsPbI_3$ perovskite calculated from EQE in **Figure S16 b**. d) Penetration depth of light into $CsPbI_3$ (and treated films) perovskite films versus photon energy.

How to measure penetration depth for perovskite thin film:

The penetration depth can the perovskite film can be calculated by the following relation;

$$l = \frac{1}{\alpha} \qquad \textbf{Eq. S8}$$

Whereas, the absorption coefficient (α) can be defined as,

$$\alpha = \frac{2.303A}{d} \qquad \textbf{Eq. S9}$$

The absorbance (A) of the light in a material has a logarithmic relationship to the transmittance (T).

$$A = -log_{10}T \qquad \textbf{Eq. S10}$$

In the experiment, absorbance (A) was calculated by transmittance (T) measurements of the thin films (measured by UV-Vis spectroscopy) as mentioned in **Figure S2a** while thickness (*d*) of the film was measured by cross-sectional SEM of the perovskite film *i.e.* ~300 nm. The photon energy at every wavelength can be described as;

$$h\nu = \frac{1240}{\lambda\,(nm)}\,eV \qquad \textbf{Eq. S11}$$

The penetration depth at every wavelength can be measured by plotting penetration depth *vs.* $h\nu$ as shown in **Fig S2 d**.

The Tauc plot method is given by **Equation S12**, in which, $(\alpha h\nu)^2$ is plotted against energy $(h\nu)$ and the linear segment is fitted to calculate the band gap (E_g)at the *x*-axis. [22,23]

$$(\alpha h\nu)^2 \propto h\nu - E_g \qquad \textbf{Eq. S12}$$

Film morphology

We noticed barely any difference in the grain size and film thickness of $CsPbI_3$ before and after TOPO treatment in **Fig. S3** . Yet, the surface morphology changed slightly due to the coverage of the TOPO layer deposited at high concentrations (particularly 20 mM). The film roughness was characterized by atomic force microscopy (AFM), with the topography images presented in **Fig. S4**. It shows a slight increase in film roughness, which is likely caused by the slight changes in surface morphology during the dripping process of solutions for the additional layers.

CsPbI3/OAI (Control)

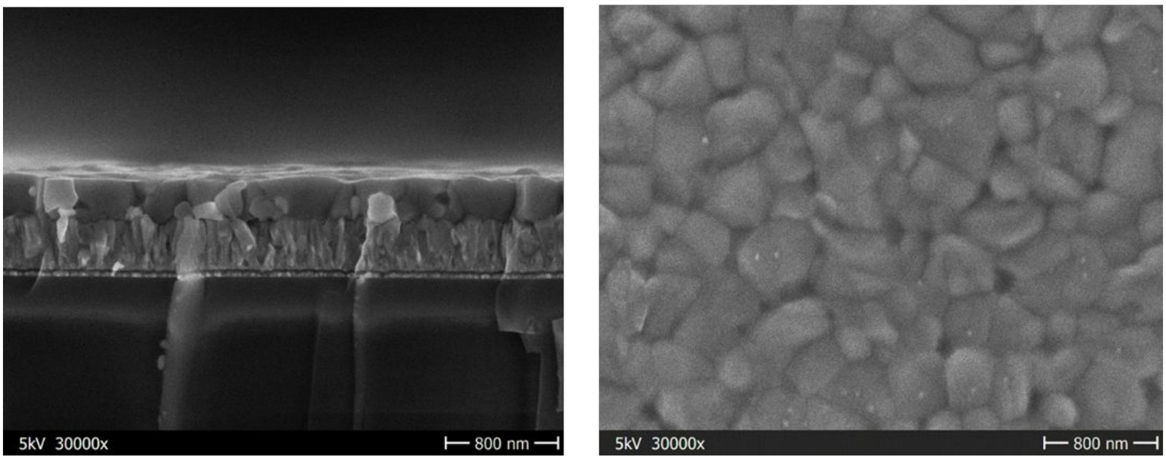

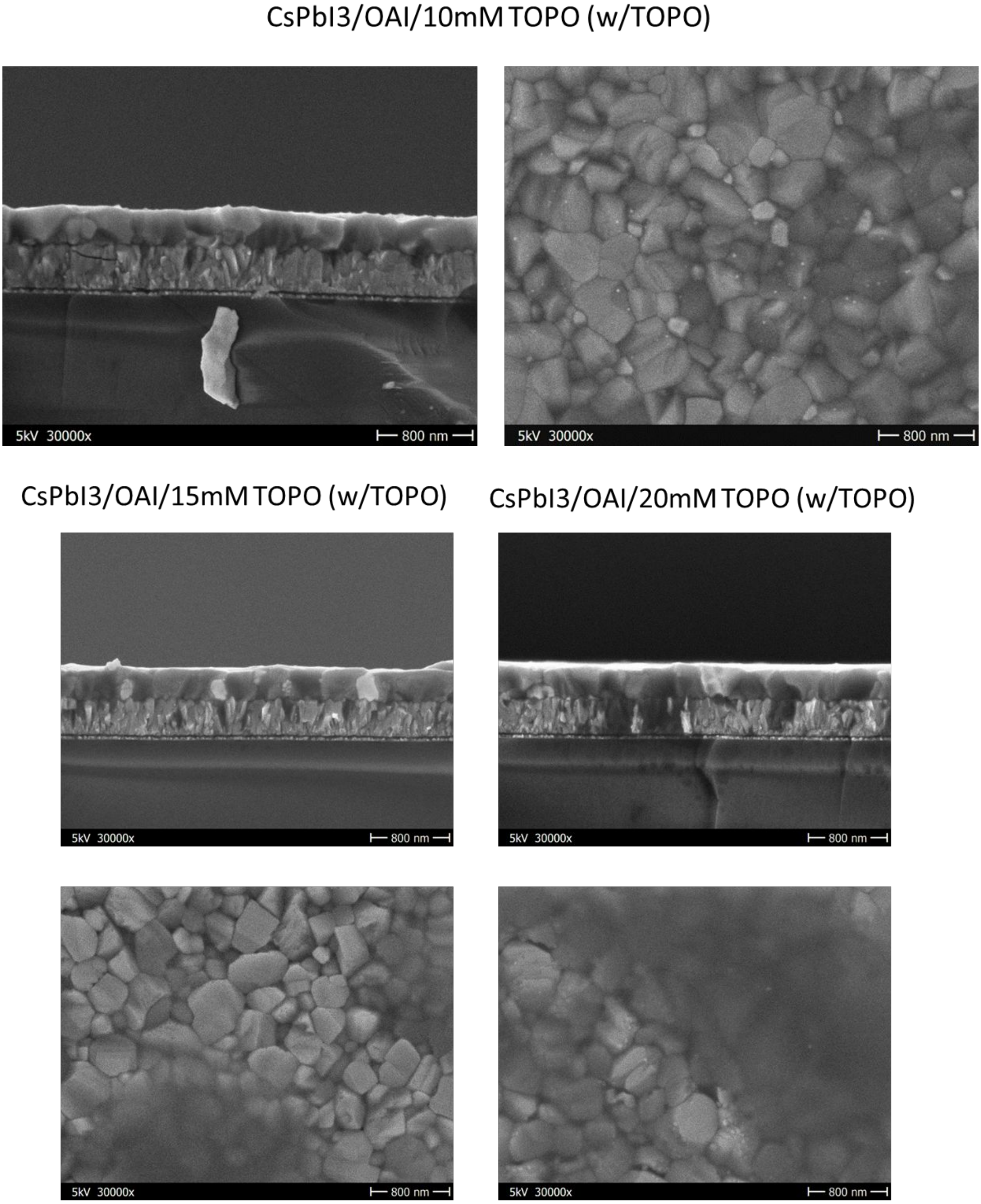

Fig. S3. SEM images (cross-section and surface morphology) of $CsPbI_3$ samples with and without TOPO treatment.

Film roughness

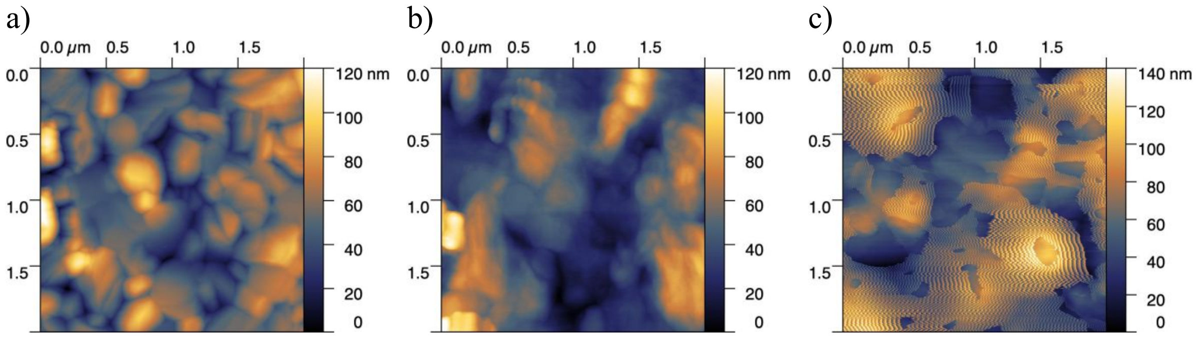

RMS = (16.6 ± 3.4) nm
Max. Height: 128.6 nm

RMS = (20.1 ± 4.3) nm
Max. Height: 128.8 nm

RMS = (24.3 ± 5.2) nm
Max. Height: 146.7 nm

Fig. S4. Topography images of a) bare $CsPbI_3$, b) control $CsPbI_3$, and c) w/TOPO, characterized by atomic force microscopy (AFM).

Note: The "lined" features in TOPO-treated samples (c) are likely to be caused by the effect of dipole force on the cantilever during the measurement.

Kelvin Probe Force Microscopy: (KPFM)

We measured AFM/KPFM on a (2 x 2) μm^2 area of $CsPbI_3$ samples without any treatment, with OAI treatment, and with OAI/TOPO treatment. The distributions of the contact potential difference (CPDs. below) were obtained from Gaussian fitting of the image histograms. In the non-treated sample, the Gaussian curve yields a full-width half-maximum (FWHM) of 240mV. As a comparison, the OAI-treated sample exhibited a decreased FWHM of 40 mV, and the OAI/TOPO-treated sample had an even lower FWHM of 30 mV. The FWHM is a figure of merit to express the variety of measured CPD values over the sample area. A decrease in the FWHM, therefore, highlights an improved homogeneity of the work function over the OAI- and OAI/TOPO-treated surfaces.

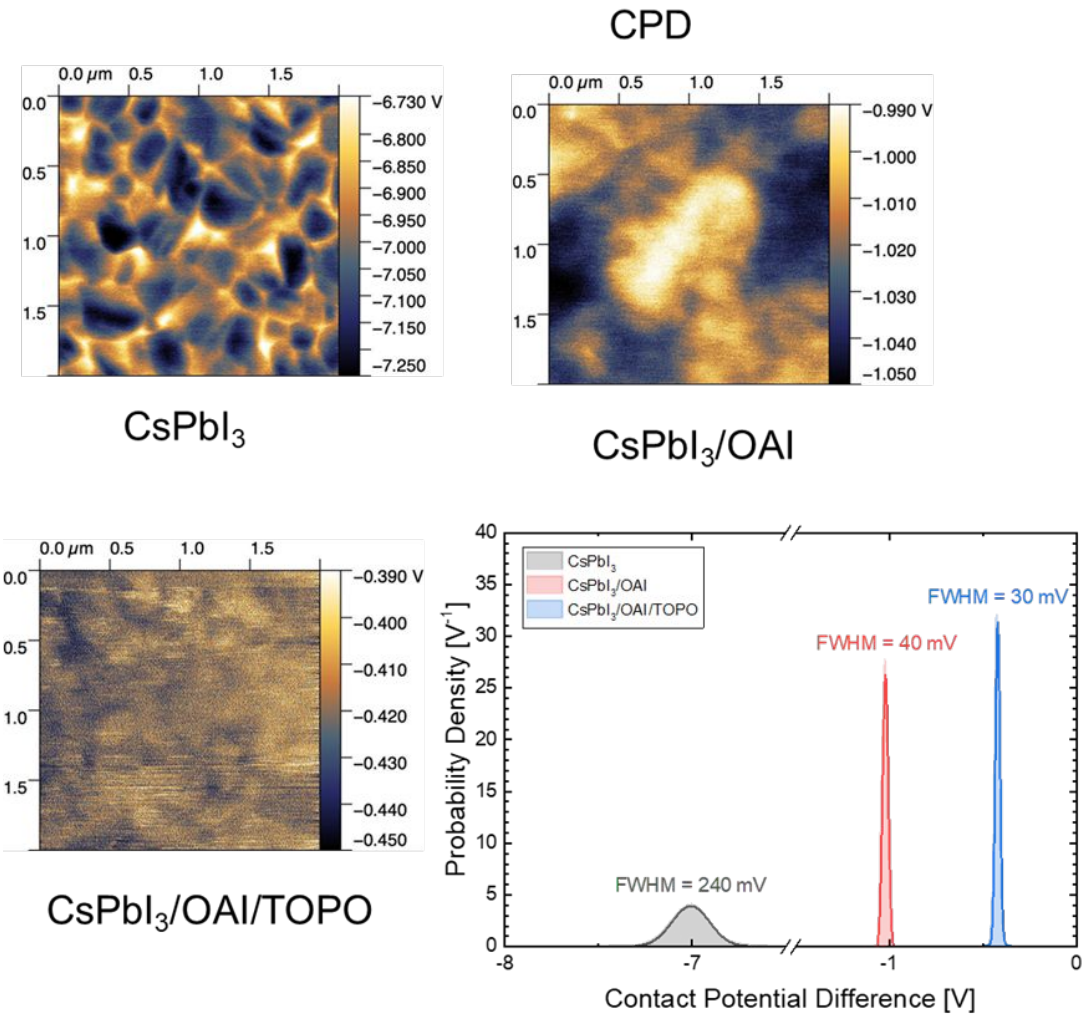

Fig S5 : Measurements of contact potential difference by KPFM for bare $CsPbI_3$, control $CsPbI_3$, and w/TOPO, characterized by AFM.

QFLS calculation

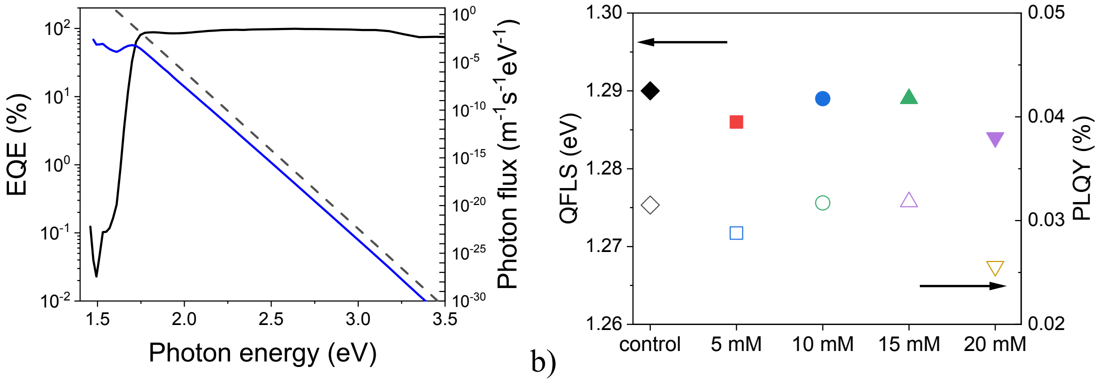

Fig. S6. (a)An example of EQE spectrum (solid black line) integrated with the black body spectrum (grey dash line) used for the illustration of the calculation of $J_{0,rad}$ (solid blue line) **Eq.S13**. b) PL quantum yield (PLQY) and quasi-fermi level splitting (QFLS) of control and TOPO-treated samples.

$$J_{0,rad} = q \cdot \int EQE_{PV}(E) \cdot \phi_{BB}(E) \cdot dE \qquad \textbf{(Eq. S13)}$$

where $J_{0,\ rad}$ is the radiative thermal equilibrium recombination current density in the dark at room temperature (at 2.5 10^{-22} Am^{-2}), q is the elementary charge, EQE (E) is the EQE spectrum as a function of photon energy (E), ϕ_{BB} is the black body spectrum, given in the following equation:

$$\phi_{BB} = \frac{1}{4\pi^2\hbar^3 c^2} \frac{E^2}{exp\left(\frac{E}{k_B T}\right) - 1} \qquad \textbf{(Eq. S14)}$$

Where h is the Plank constant, c is the speed of light in vacuum, and k_BT is the thermal energy at room temperature.

QFLS is calculated from the following equation:

$$QFLS = \frac{k_B T}{q} \ln\left(\frac{J_G}{J_{0,rad}} + 1\right) + \frac{k_B T}{q} \ln(PLQY) \qquad \textbf{(Eq. S15)}$$

Where J_G is the photogenerated current density, taken as J_{sc} measured from *J-V* curves.

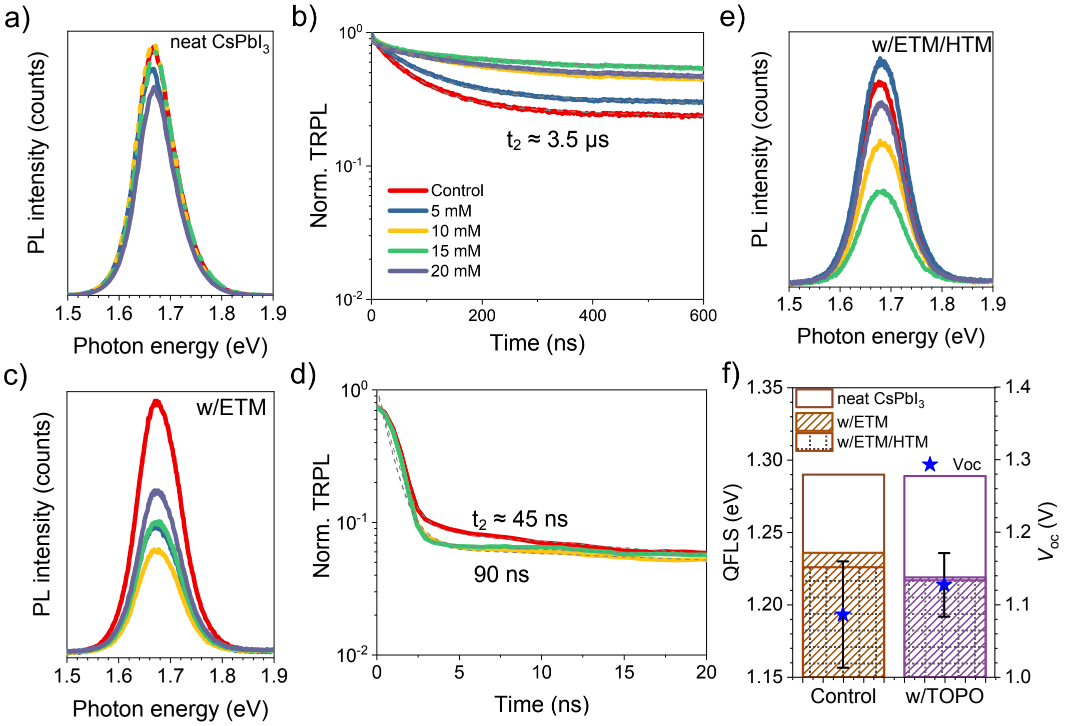

Fig. S7 a) Steady-state and b) time-resolved photoluminescence of neat perovskite films deposited on quartz for control (red line), 5 mM (steal blue line), 10 mM (yellow line), 15 mM (green line) and 20 mM (lavender line) TOPO treated samples at a fluence of XZ nJcm^{-2} using a XZ nm laser. c) Steady-state and d) time-resolved photoluminescence of perovskite films with ETM for control (red line), 5 mM (steal blue line), 10 mM (yellow line), 15 mM (green line) and 20 mM (lavender line) TOPO treated samples. e) Steady-state photoluminescence of perovskite films with ETM and HTM for control (red line), 5 mM (steal blue line), 10 mM

(yellow line), 15 mM (green line), and 20 mM (lavender line) TOPO treated samples. f) QFLS for neat perovskite (hollow bars), with ETM (bars with stripes), and with ETM and HTM (bars with dashed square lines) for control and 10 mM TOPO treated samples.

Perovskite films deposited on top of ETM were characterized with PL as well (**Fig. S7 c.**). Faster quenching is observed for all TOPO-treated samples. In general, faster quenching in PL signals can be caused by increased trap states or faster charge extraction at the interface with the charge selective layer. In our case, we believe it comes from faster electron extraction at the interface with ETM with the presence of TOPO. This is confirmed by TrPL in **Fig. S7 d.** It shows that TOPO-treated samples have a t_2 longer than that of the control, while trap states lead to a smaller t_2. **Fig. S7 e** presents the steady-state PL of samples with both ETM and HTM. For 10 mM and 15 mM TOPO-treated samples, stronger quenching is observed. The quasi-fermi level splitting (QFLS) for control and 10 mM TOPO treated samples are summarized in **Fig. S7 f**. It shows that a smaller difference between QFLS and V_{oc} (averaged from over 40 solar cells) is seen in TOPO-treated samples.

Fitting of TRPL

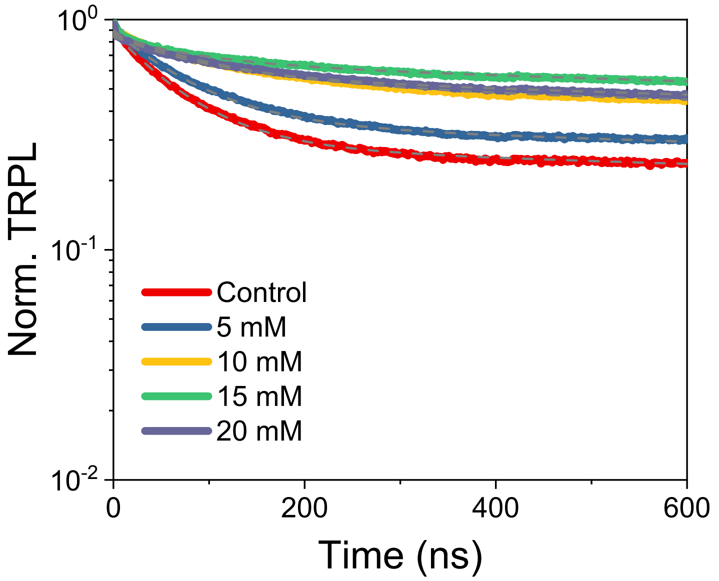

Fig. S8. TrPL fitted with a bi-exponential equation **S16**.

Here we observed that our TRPL data in **Fig. 1b**, **Fig. S7 b,d,** and **Fig.S8** showed a bi-exponential decay instead of a mono-exponential one. It indicates that the bimolecular electron-hole recombination is dominating in the PL decay of our samples while the trap-related monomolecular recombination lifetime is rather long, up to a microsecond.[24,25,26] Thus, we fitted the spectra with a bi-exponential equation given in the following:

$$y = A_1 * \exp\left(\frac{-x}{\tau_1}\right) + A_2 * \exp\left(\frac{-x}{\tau_2}\right) \qquad \textbf{(Eq. S16)}$$

Table S2. Fitted parameters of TRPL spectra in **Fig. 1b** and **Fig. S7 b**,**d.**

Neat perovskite film	A_1	A_2	t_l (ns)	t_2 (ns)
Control	0.6	0.28	68	3500
5 mM TOPO treatment	0.55	035	77	3500
10 mM TOPO treatment	0.34	0.53	110	3500
15 mM TOPO treatment	0.2	0.64	92	3500
20 mM TOPO treatment	0.29	0.56	90	3500
w/ ETM	0.96	0.09	0.8	45
10 mM TOPO treatment	0.95	0.07	0.75	90
15 mM TOPO treatment	0.95	0.07	0.75	90

Comparing the τ_1 extracted from **Fig. S7 b** and **d**, the value is dropped by approximately one order of magnitude, indicating the τ_1 related decay happened much faster in the samples with ETM. Meanwhile, the weight (A_1) of the first exponential decay over the whole PL decay curve increased in **Fig. 1b**, indicating that τ_1 related decay contributes to a large portion of the PL decay. We believe the difference comes from the presence of the electron contact layer in samples presented in **Fig. S7 d**. For neat perovskite films without ETM or HTM, the PL decay comes from either the trap-related monomolecular recombination or the electron-hole-related bimolecular recombination. In our trPL data, the τ_1 reflects the decay due to the electron-hole-related bimolecular recombination while the τ_2 reports the decay coming from the trap-related monomolecular recombination. The long monomolecular lifetime of up to 3.5 μs extracted from our work agrees with the literature. [24,25,26]

With the ETM adjacent layer, the photo-generated electrons and holes in the perovskite film can experience bimolecular recombination and trap-inducted monomolecular recombination, and additionally, electrons be extracted at the interface. Each of the three paths will lead to a decay in the PL signal. It is likely that for perovskite with ETM, τ_1 relates to the electron extraction reduced PL decay because we did not observe such a fast decay in neat perovskite films. τ_2 might connect to the trap-related monomolecular recombination or the electron-hole bimolecular recombination. If it reflected the decay due to monomolecular recombination, then the value is reduced by nearly two orders of magnitude compared to the neat perovskite samples. This would mean a large increase in trap densities because of the introduction of the ETM adjacent layer. However, the contour plots of the transient SPV in **Fig. S13** reflect no formation of trap states below the bandgap of $CsPbI_3$ perovskite deposited on TiO_2-ETM. Thus, we believe that τ_2 is not likely related to the trap-related monomolecular recombination. Here we assign it to the decay process caused by electron-hole bimolecular recombination. The value falls in the similar range of the τ_1 in neat perovskite samples.

XPS spectra of Pb 4f and I 3d

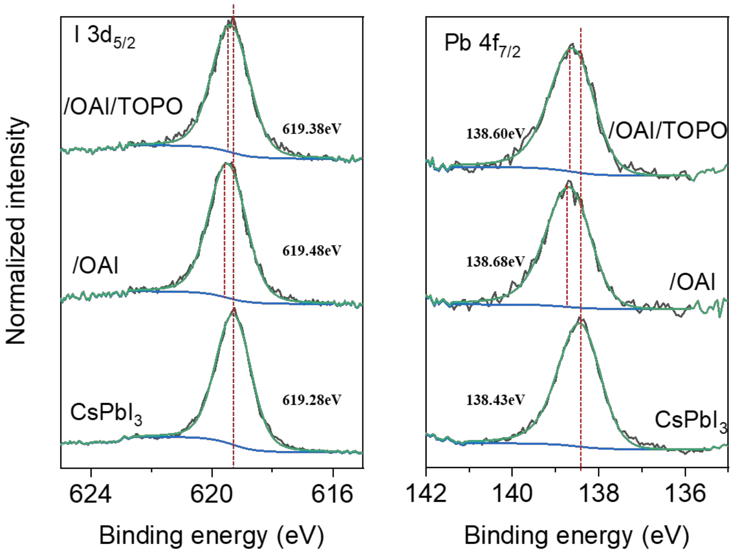

Fig. S9. XPS of a) Pb $4f_{7/2}$ and b) I $3d_{5/2}$ core-shell spectra of bare $CsPbI_3$, control sample and w/TOPO. Dashed lines in black are fitted curves and backgrounds with guidelines in grey.

Table S3. Binding energy (in eV) values I3d5/2 and Pb4f7/2 for $CsPbI_3$ bare, $CsPbI_3$+OAI (Control) and $CsPbI_3$/OAI/TOPO (w/TOPO)

Sample	I3d5/2 (eV)	Pb4f7/2 (eV)
$CsPbI_3$	619.28	138.43
$CsPbI_3$/OAI (Control)	619.48<br>(Δ0.20) ~200meV	138.68<br>(0.25) ~250meV
$CsPbI_3$/OAI/TOPO (w/TOPO)	619.38	138.60

XPS spectra of P 2s and O 1s

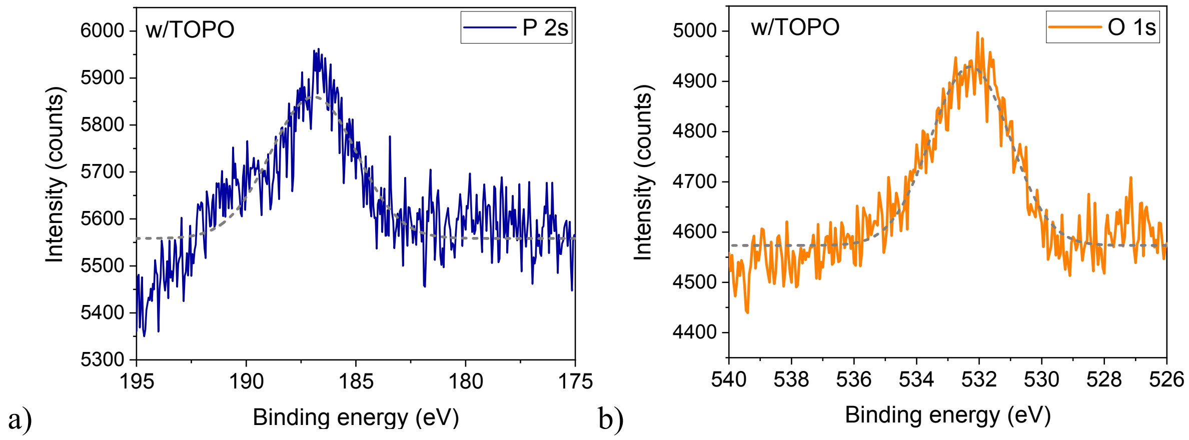

Fig. S10. XPS of a) P 2s and b) O 1s core-shell spectra of $CsPbI_3$ sample with TOPO treatment (dash lines are the Gaussian fitting of each peak).

Valence spectra on logarithmic intensity scale

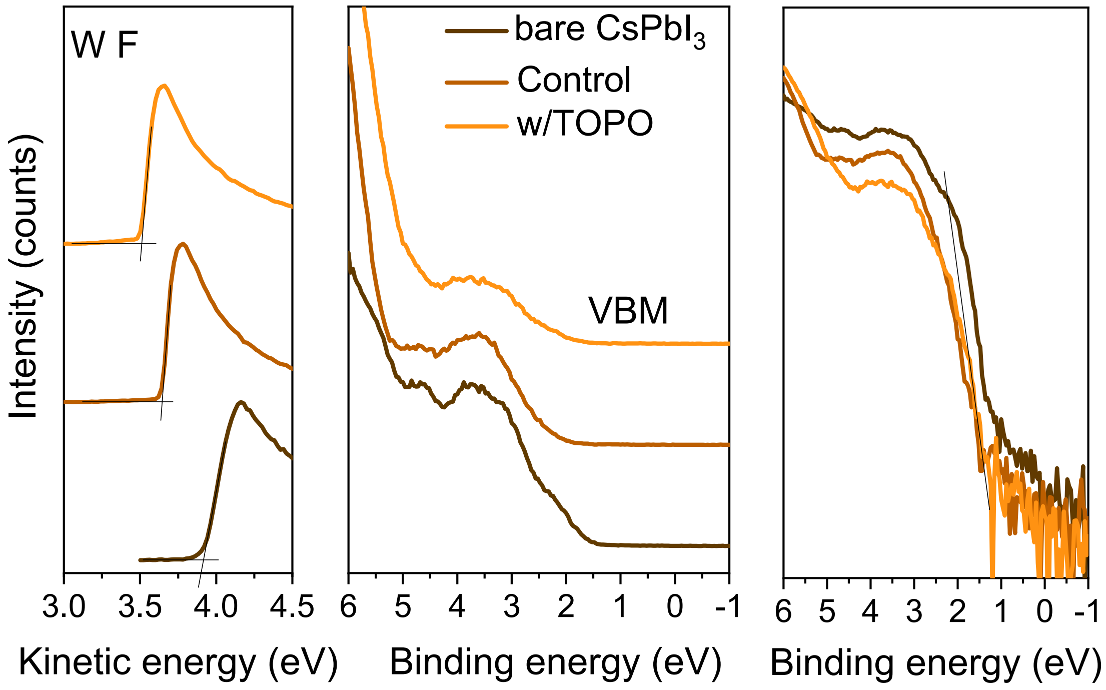

Fig. S11. Secondary electron cut-off (left panel), valence spectra (middle panel), and zoomed valence spectra on logarithmic intensity scale (right panel) of bare $CsPbI_3$, control $CsPbI_3$, and w/TOPO.

Table S4. Work function (Φ), ΔE, and VBM of the following samples extracted from UPS measurement.

Samples stack	Φ (eV)	ΔE (eV)	VBM (eV)	*CBM (eV)
bare $CsPbI_3$	-3.91	1.34	-5.25	-3.54
control $CsPbI_3$	-3.64	1.54	-5.18	-3.47
w/TOPO	-3.51	1.44	-4.95	-3.24
control/spiro-OMeTAD-dark	-4.51	0.49	-5.00	-2.08
w/TOPO/spiro-OMeTAD-dark	-4.39	0.56	-4.95	-2.03
control/spiro-OMeTAD-light	-3.85	1.15	-5.00	-2.08
w/TOPO/spiro-OMeTAD-light	-3.77	1.18	-4.95	-2.03

*Notes: CBM was calculated from the VBM-E_g (optical bandgap, calculated from the Tauc plot given in **Fig. S2 b** for $CsPbI_3$ (*i.e.* 1.70 eV). The optical bandgap of spiro-OMeTAD of 2.92 eV was taken from our previous publication.[27]

Work function shift

We further confirmed the shift in WF in our samples using the Kelvin probe. **Fig. S12a** shows the average WF of these samples given with an error bar. We observe that bare $CsPbI_3$ film has an average WF of 4.35 eV, reduced to 4.18 eV for control samples, and further reduced to 3.9 eV after TOPO treatment. The energetic level scheme is presented in **Fig. S12b**, with values summarised in **Table S5**. The steady decrease in WF agrees well with the discussion on UPS data in the main context. The difference in absolute values of WF between the Kelvin probe and UPS characterization could be caused by the slight sample variation or the different measurement environments of the two techniques. The slightly lower values of WF probed by UPS can also be explained by the illumination of samples by the Ultra-violate light during the measurement, which increased sample conductivity and occupied free states.

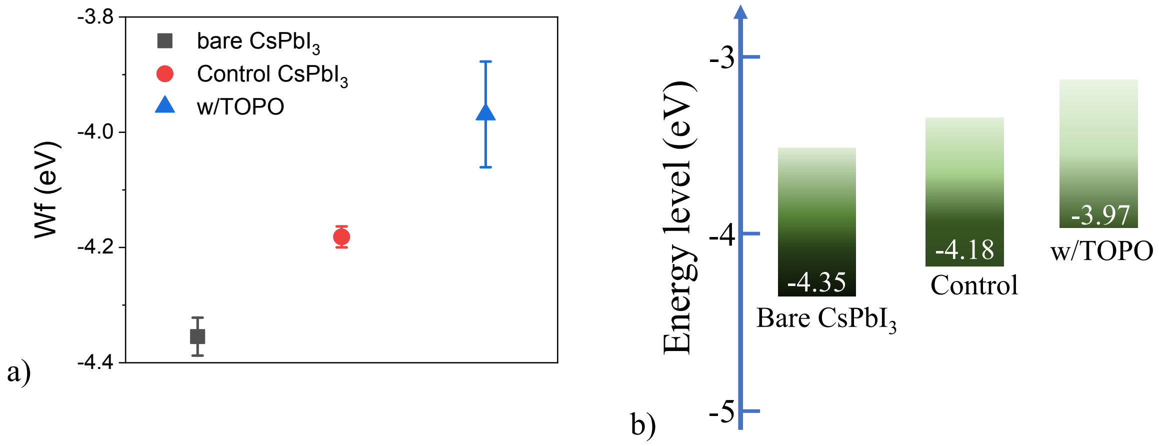

Fig. S12. a) Work function (Φ) of bare $CsPbI_3$ (black square), control $CsPbI_3$ (red sphere), and w/TOPO (blue triangle) with error bars (standard deviation, in unit of eV), measured by Kelvin probe technique. b) energy level diagram of these samples, taking the average value plotted in a).

Table S5. The work function (Φ) of the following samples was measured by the Kelvin probe.

Samples stack	WF Right corner (eV)	WF Left corner (eV)	WF Middle part (eV)	WF Average (eV)	Standard deviation (eV)
Bare $CsPbI_3$	-4.333	-4.401	-4.330	-4.355	0.033
Control $CsPbI_3$	-4.205	-4.178	-4.161	-4.183	0.018

w/TOPO	-4.098	-3.916	-3.893	-3.969	0.092

Influence of substrates characterized by tr-SPV

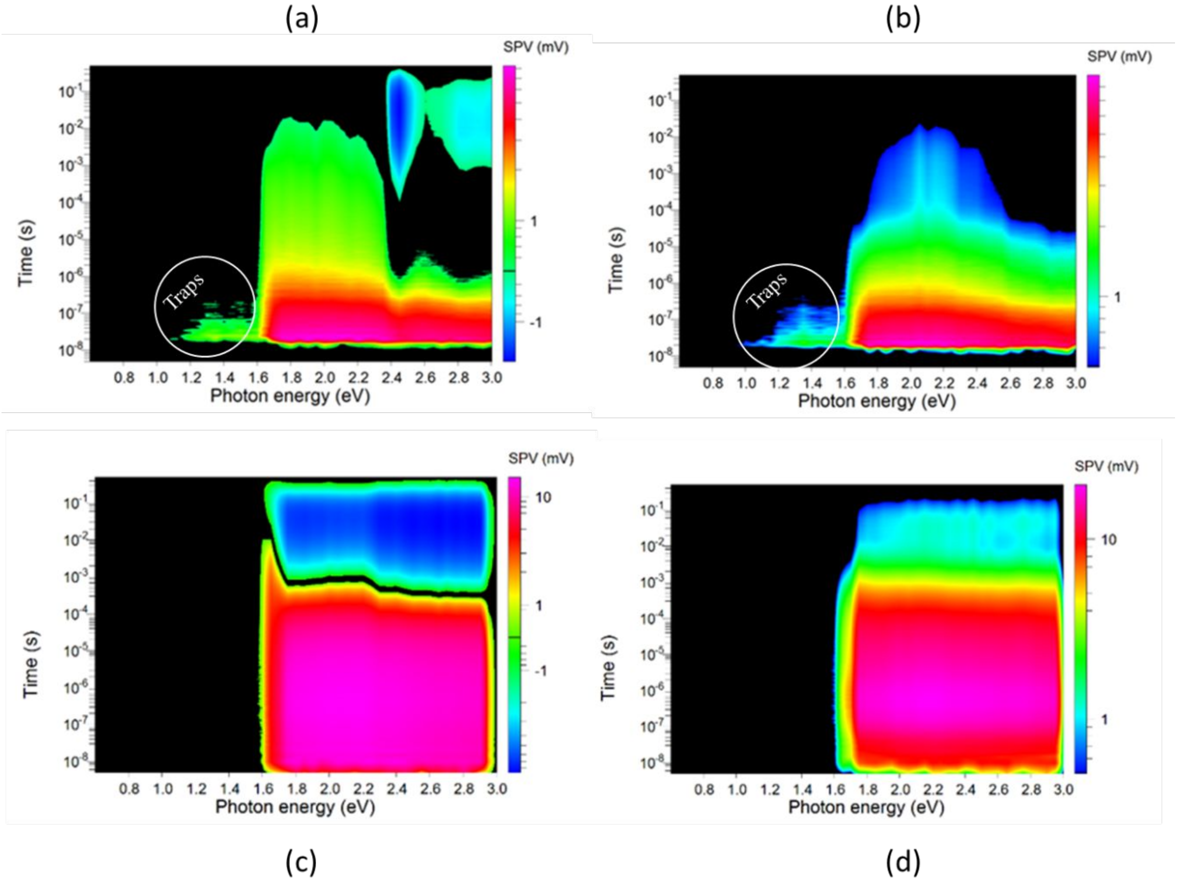

Fig. S13. Contour plots of the transient SPV illuminated by different light energies of a) glass/$CsPbI_3$/OAI (Control), b) glass/$CsPbI_3$/OAI/TOPO, c) glass/TiO_2/$CsPbI_3$/OAI(Control), d) glass/TiO_2/$CsPbI_3$/OAI/TOPO.

Figure S13 shows SPV signals as a function of photon energy and time. Whereas SPV amplitude is presented as a color code in the right corner. The SPV signals are measured with light excitation by photon energy in the range of 0.7-3 eV. The excitation of the trap by light leads to charge separation. The signal below the band gap corresponds to the activation energy of the trap. SPV signal is induced by the diffusion of the excited free carrier out of a trap. The activation energy is the lowest photon energy that we observe in the SPV signals. A similar approach has been used, in previous studies [28,29,30] to find the activation energy.

Transient SPV at 0.1 sun and 10 suns

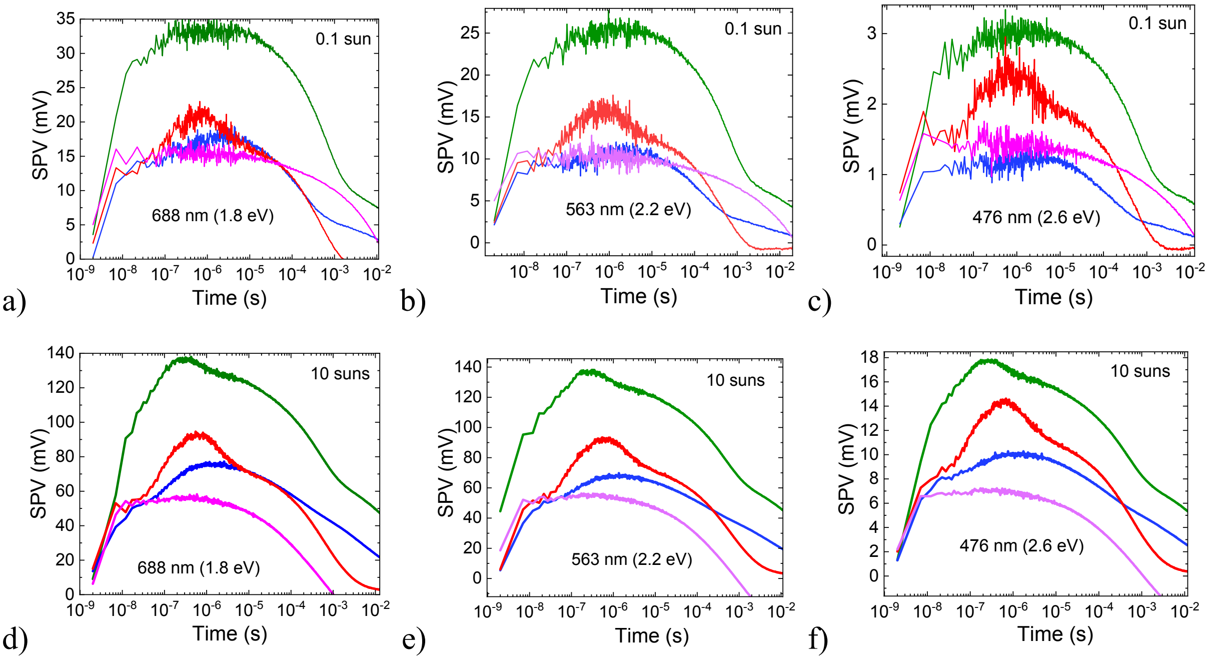

Fig. S14. Transient SPV of control (pink line), w/TOPO (red line), control with spiro-OMeTAD (blue line), w/TOPO/spiro-OMeTAD (green line) at a) 1.8 eV, 0.1 sun, b) 2.2 eV, 0.1 sun, c) 2.6 eV, 0.1 sun, d) 1.8 eV, 10 suns, e) 2.2 eV, 10 suns, and f) 2.6 eV, 10 suns.

Table S6. Additional constants for simulations

Where N, P, C_b, σ_e, σ_h, v_e, and v_h are photo-induced electron concentration, photo-induced hole concentration, radiative recombination constant, electron capture-cross section of trap, hole capture cross-section of trap, thermal velocity of electron and hole, respectively. ε_{per}, $\varepsilon_{ETM,}$ and ε_{HTM} are dielectric constants. τ_{HTM} and τ_{ETM} are lifetimes in HTM and ETM. The concentration of free carriers (N, P) was calculated according to the fluence of 0.010 µJ/cm^2. Constants C_b, v_e, v_h, and $\boldsymbol{\varepsilon_{per}}$, were adapted from our previous study [2]. Dielectric constants ε_{ETM} and ε_{HTM} were adapted from literature [31,32]. Constants τ_{HTM}, τ_{ETM}, K_{eETL}, σ_e, and σ_h are fitted directly and are in agreement with previous reports[2, 32,33].

**N (P), cm^{-3}**	**C_b, cm^{3}s^{-1}**	**K_{eETL}, 10^7 s^{-1}**	**σ_e, cm^2**	**σ_h, cm^2**	**v_e, cms^{-1}**
1.1×10^{15}	5×10^{-10}	1.2-1.8	10^{-13}	4×10^{-14}	3×10^{7}
v_h, cm s^{-1}	***ε_{per}***	***ε_{ETM}***	***ε_{HTM}***	***τ_{HTM} s***	***τ_{ETM} s***
3×10^{7}	18	3	3	$2\text{-}5\times10^{-5}$	$2\text{-}4\times10^{-4}$

Transient SPV fitting results

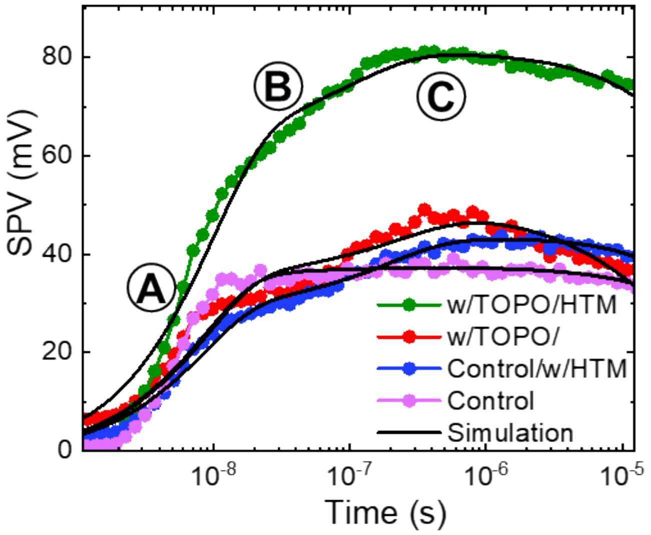

Fig. S15. Transient SPV of control sample (pink line), w/TOPO (red line), control with spiro-OMeTAD (blue line), w/TOPO/spiro-OMeTAD (green line). Fitted results are given in black lines.

Table S7 Main fitting constants of simulation. $N_{non\text{-}rad}$ – concentration of non-radiative recombination defects (both surface or bulk), K_h – hole injection rate, K_e – electron injection rate, K_{hb} – hole reinjection rate, and K_{eb} – electron reinjection rate. *STD* is the average standard deviation of the fit from the experimental SPV signal.

Interface	K_h, s^{-1}	K_e, s^{-1}	K_h/K_e	$N_{non\text{-}rad}$, cm^{-3}	*STD*, %
w/TOPO/spiro-OMeTAD	8×10^6	10^6	8	7.7×10^{13}	11.6
control/ spiro-OMeTAD	4.4×10^6	3.6×10^6	1.2	9.4×10^{13}	6
w/TOPO	3.9×10^6	10^6	3.9	8.8×10^{13}	15.2
Control	1.5×10^6	1.1×10^6	1.3	9.1×10^{13}	8

Champion *J-V* of $CsPbI_3$ solar cells with TOPO at varied concentrations

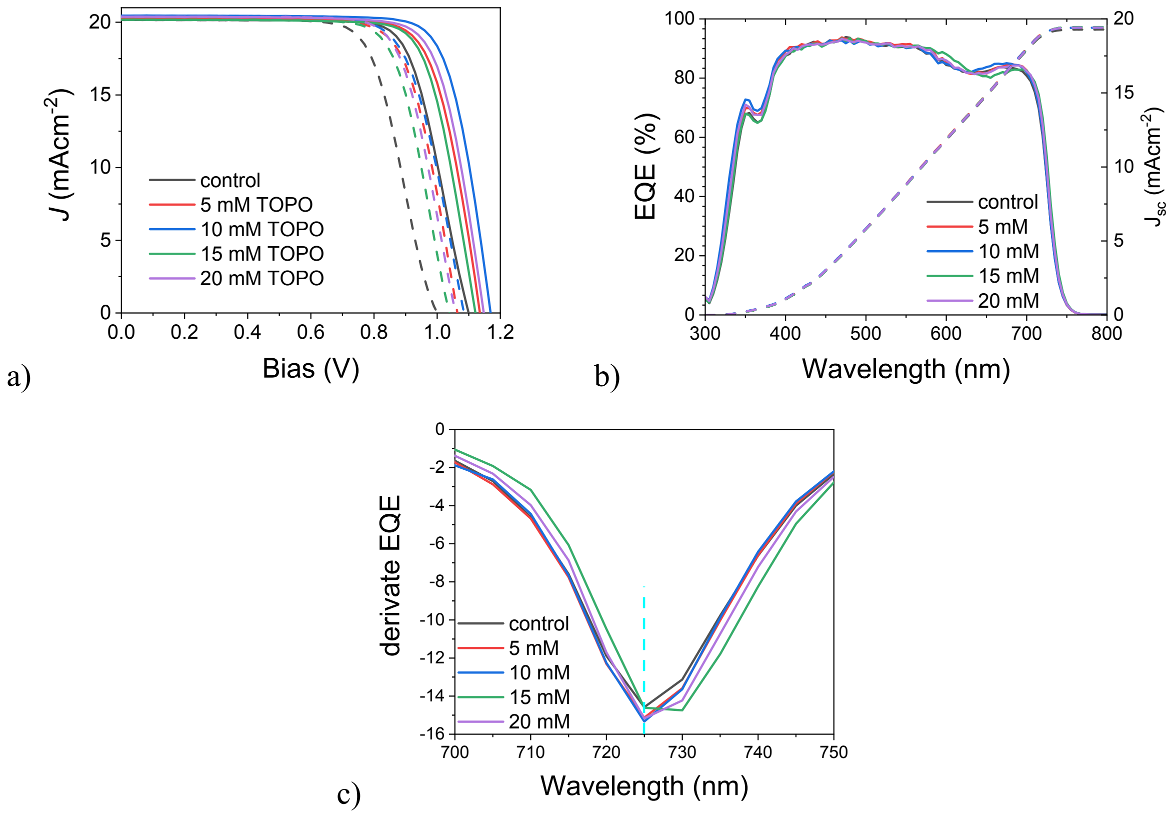

Fig. S16. a) *J-V* curve of the champion devices for $CsPbI_3$ with and without TOPO treatment, measured at 1 Sun AM1.5G at room temperature inside the nitrogen-filled glovebox at the scan rate of 200 mV/s for both forward (dash line) and reverse (solid line) scans. b) EQE spectra of champion devices with the integrated J_{sc} from EQE spectra overplotted on the right y-axis. c) Absorption onset of $CsPbI_3$ solar cells extracted from derivated EQE spectra.

Table S8. Photovoltaic parameters summary for champion devices of $CsPbI_3$ perovskite.

	J_{sc} ($mAcm^{-2}$)	V_{oc} (mV)	*FF* (%)	PCE (%)	
Control	20.2	1100	74.7	16.6	re
	20.2	1000	70.5	14.3	fw
5 mM TOPO	20.3	1135	76.3	17.6	re
	20.3	1064	74.2	16.0	fw
10 mM TOPO	20.5	1169	78.0	18.7	re
	20.5	1085	73.1	16.2	fw
15 mM TOPO	20.2	1121	76.6	17.3	re
	20.2	1038	74.4	15.6	fw
20 mM TOPO	20.4	1147	76.9	18.0	re
	20.4	1058	74.3	16.0	fw

Notes: "re" and "fw" indicate the reverse scan (bias sweeps from V_{oc} to J_{sc}) and forward scan (bias sweeps from J_{sc} to V_{oc}.), respectively.

Statistic *J-V* data

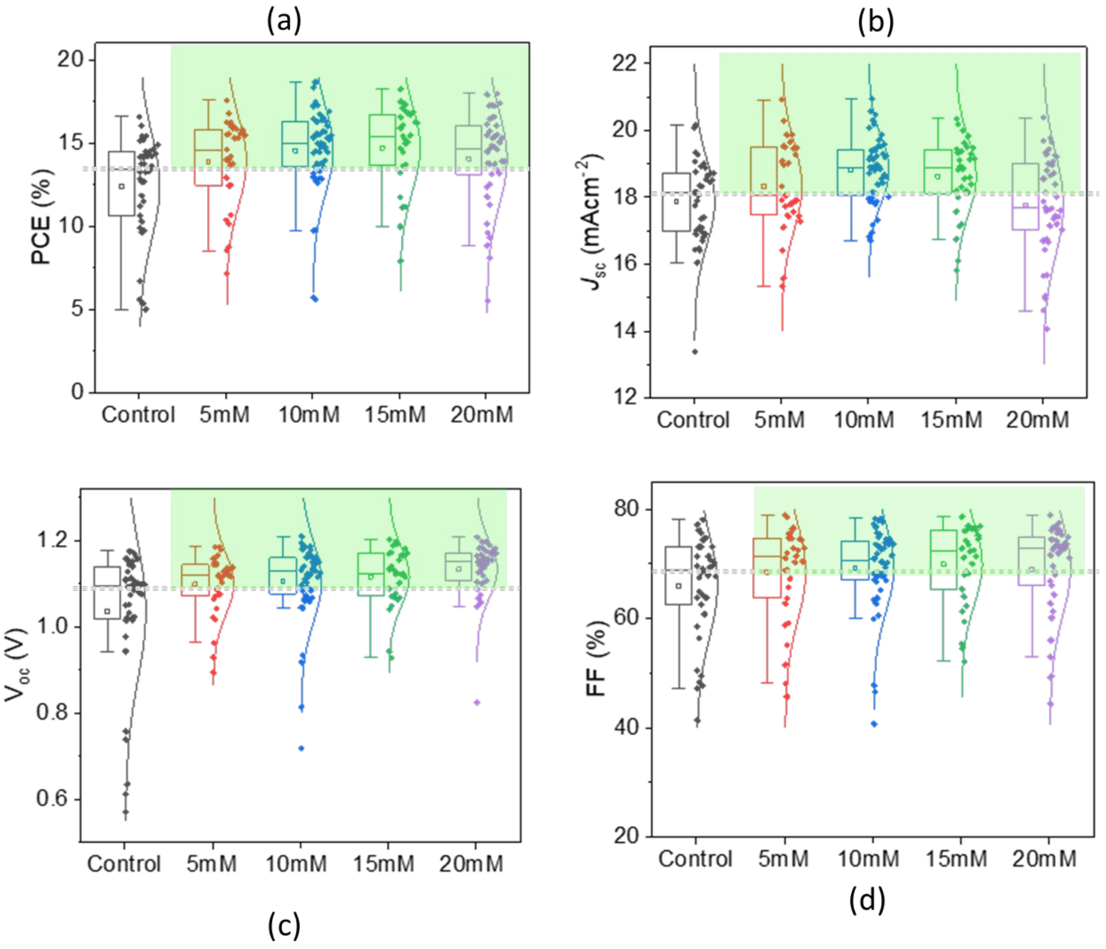

Fig. S17. Box charts of a) PCE, b) J_{sc}, c) $V_{oc,}$ and d) *FF* of more than 40 solar cells ("shunted/dead" solar cells data are removed and not included). The data (shaded area) shows that there is an improvement in PCE and other parameters for TOPO-treated samples.

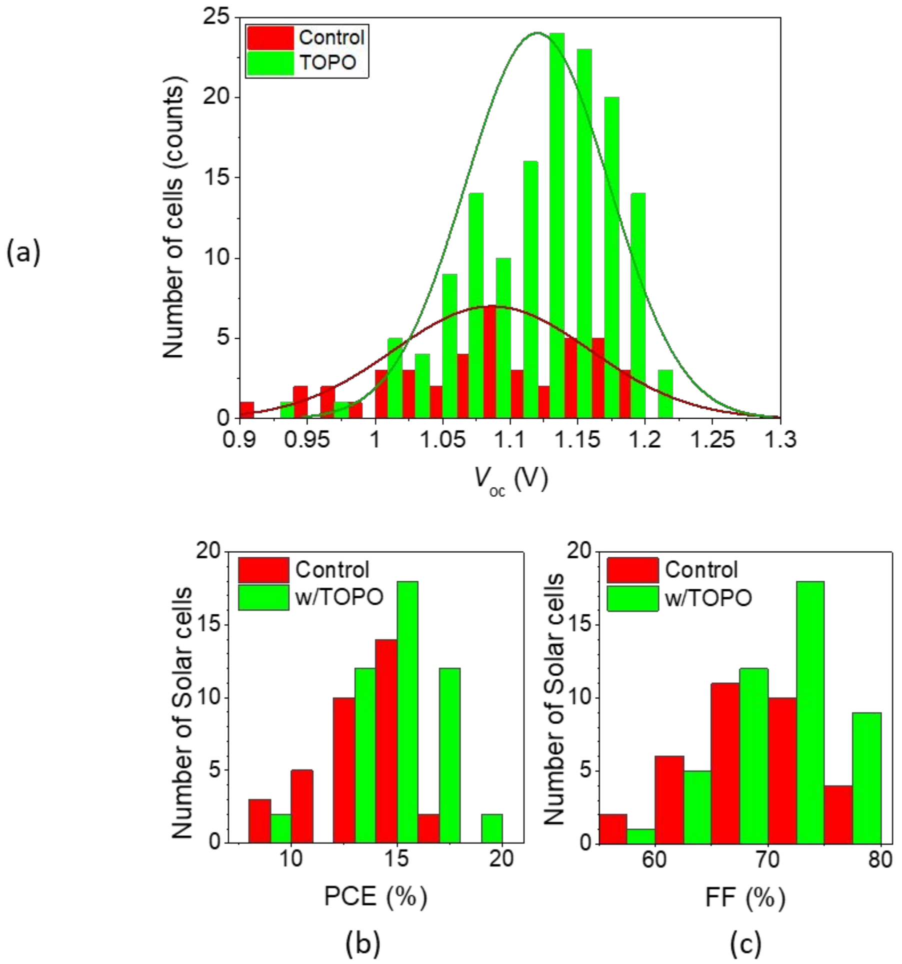

Fig. S18. Distribution analysis of a) V_{oc} b) PCE and c) *FF* of more than 70 solar cells for control and TOPO ("shunted/dead" solar cells data are removed and not included).

Champion V_{oc} devices

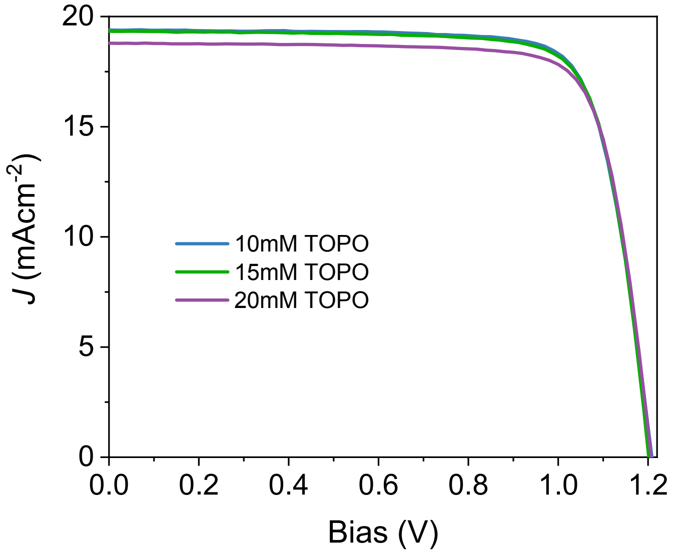

Fig. S19. *J*-*V* curves of solar cells showing V_{oc} of over 1.2 V.

Long-term stability data-I

The analysis is based on four individual solar cells for each condition and each solar cell substrate carries six pixels. We selected the one that presented the best efficiency and stability. Ageing was conducted with four substrates (samples) for each type of solar cell (24 pixels per condition). We refer to this selection method as the "best PCE+stability filter". In the following, those champion pixels are shown.

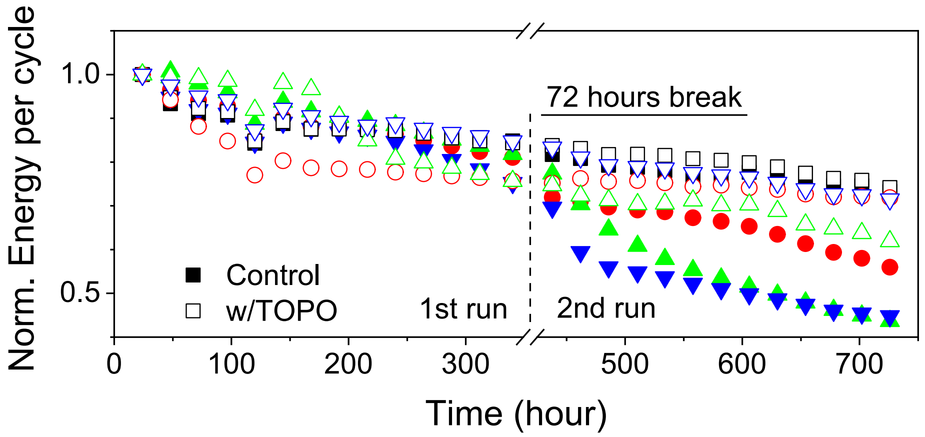

a)

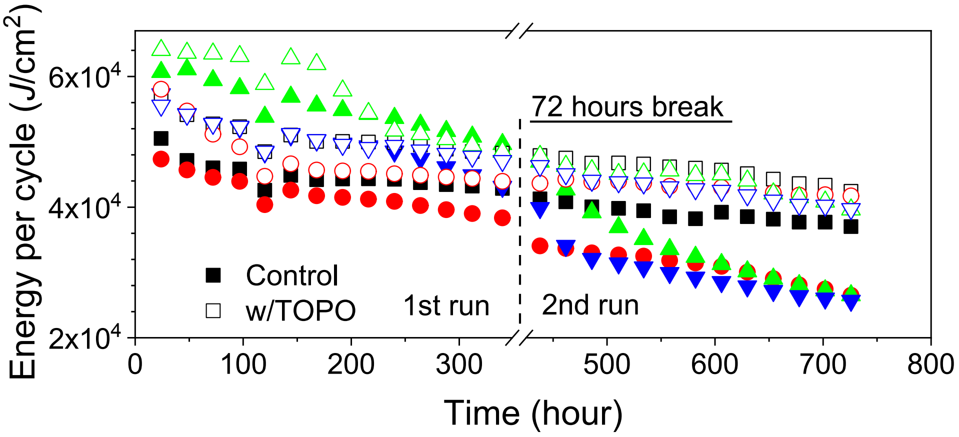

b)

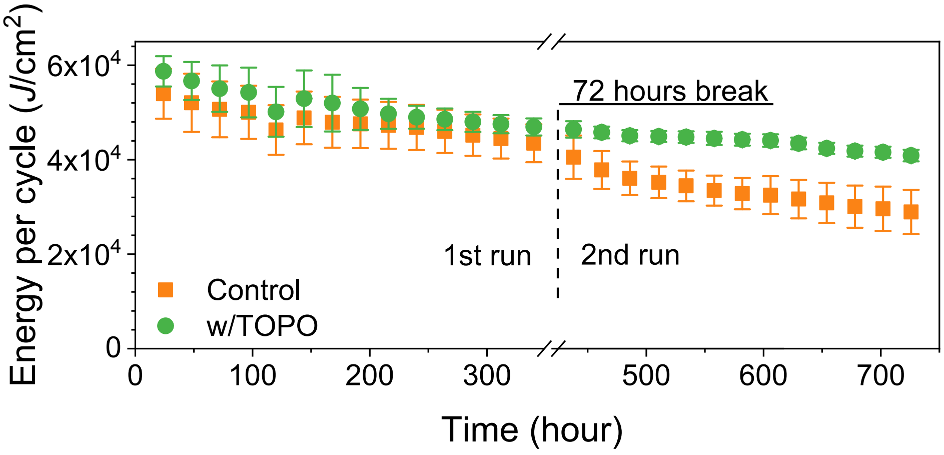

c)

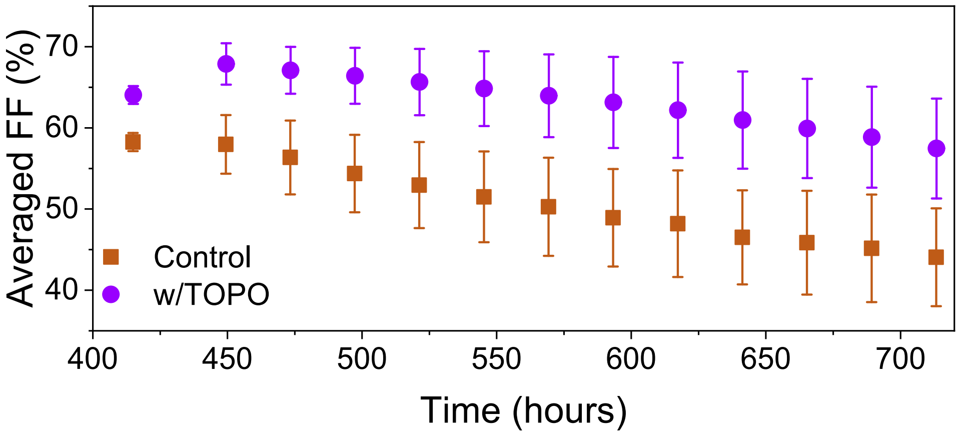

d)

Fig. S20. a) normalized and b) absolute energy per cycle of four individual solar cells, c) absolute energy per cycle on average of four individual solar cells, and d) averaged FF evolution of four solar cells in the long-term stability test from the 420th hour, *i.e.* the second run.

Long-term stability data-II (Figure S21 a-c)

An analysis based on the distribution of maximum PCE. From the overall 24 solar cells (six on each substrate), we selected 18 out of them following the protocol given below. We refer to this method as the "PCE distribution filter".

a) Distribution of maximum PCE for the control and TOPO-treated samples. Maximum PCE was taken from the first illumination cycle.

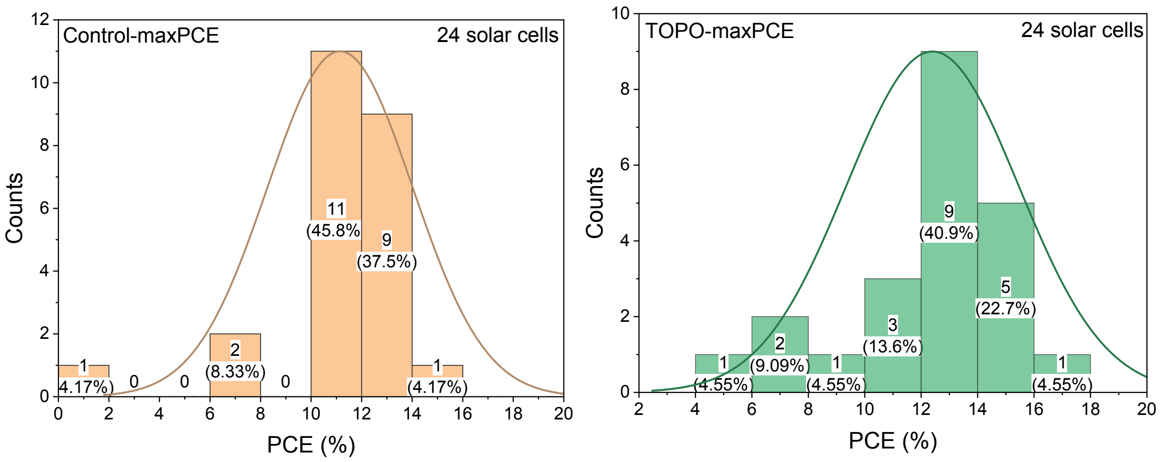

b) We selected the solar cells that present an efficiency within the top 75% that covers pixels from each substrate. For the control, it includes six pixels from sample Z11, five pixels from sample Z16, three from Z8, and four from Z10. For the TOPO-treated samples, it includes five pixels from Z7, four pixels from Z2, four pixels from Z6 and five pixels from Z13. Overall, 18 pixels out of 24 pixels are selected. Meanwhile, all the selected solar cells possess an efficiency of over 10%. The stability data with cycled illumination for the first round are given in the following.

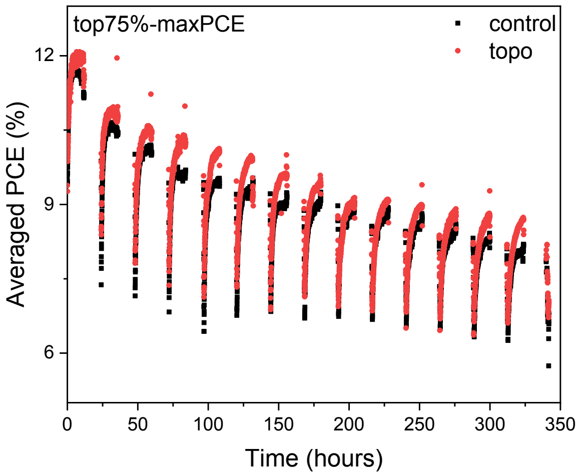

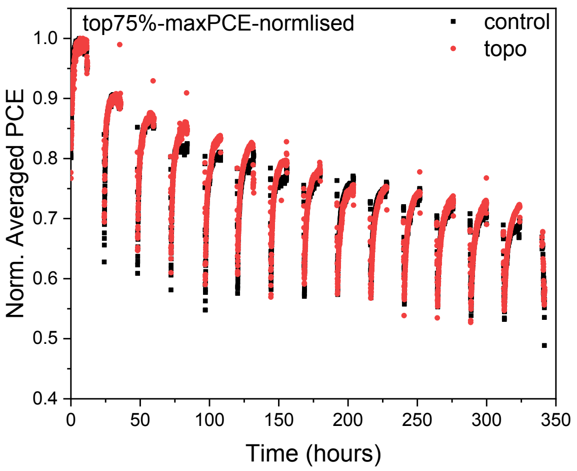

c) In the second round, three pixels were taken out of 18 pixels because of their low efficiency. For the control samples, we have four pixels from sample Z11, four from Z16, three from Z8, and four from Z10. For the TOPO-treated samples, we have four pixels from Z7, three from Z2, four from Z6, and four from Z13. So, we have 15 pixels for the stability data for each condition. The results are as follows.

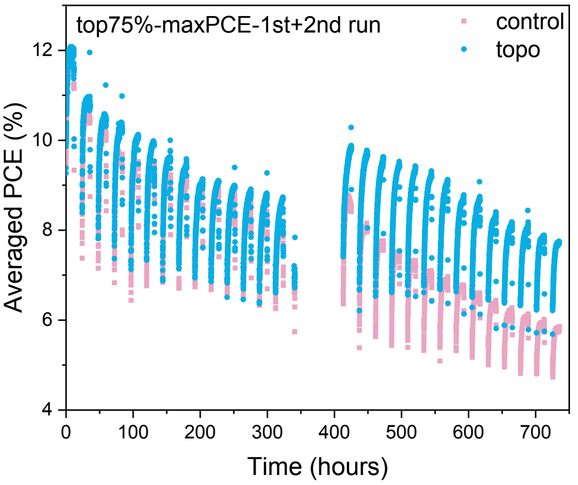

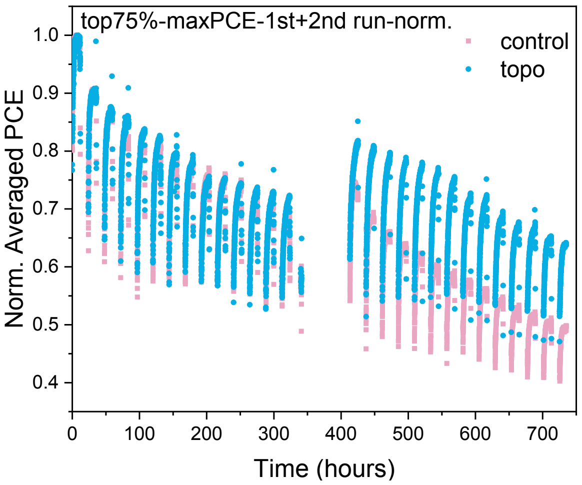

The superior stability in topo-treated samples becomes dominant in the second-round experiment. This conclusion we reached based on the "PCE distribution filter" agrees well with what we discovered with the "best PCE+stability filter".

Long-term stability data (III)

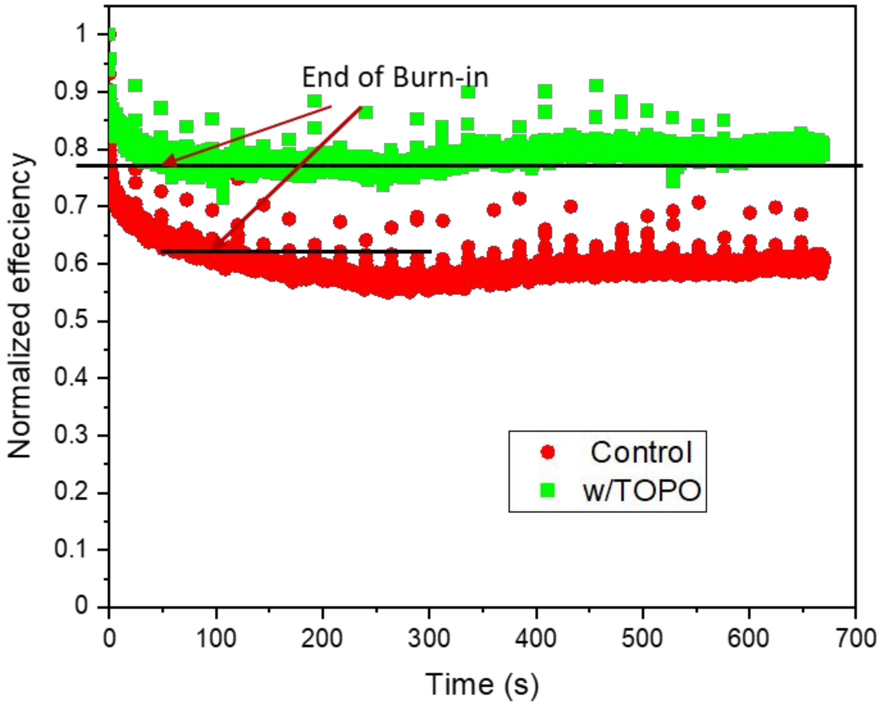

Fig. S22. Device Stability: MPP tracking for control and TOPO-treated samples for 700 hours

To further, compare the stability, we have conducted constant illumination aging testing for up to 700 hours. **Fig. S22** shows that both types of devices undergo burn-in initially and then get stabilized. Compared to the control, TOPO-treated devices tolerate the efficiency for a long time.

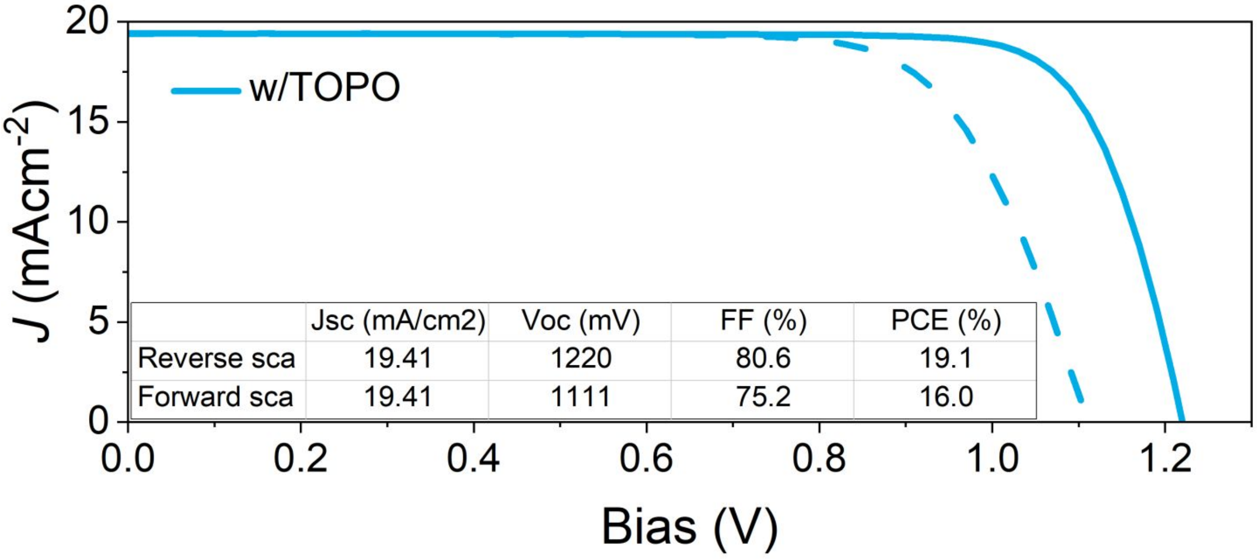

Fig. S23. Champion *J-V* of TOPO-treated perovskite solar cells with PCE of 19.1%.

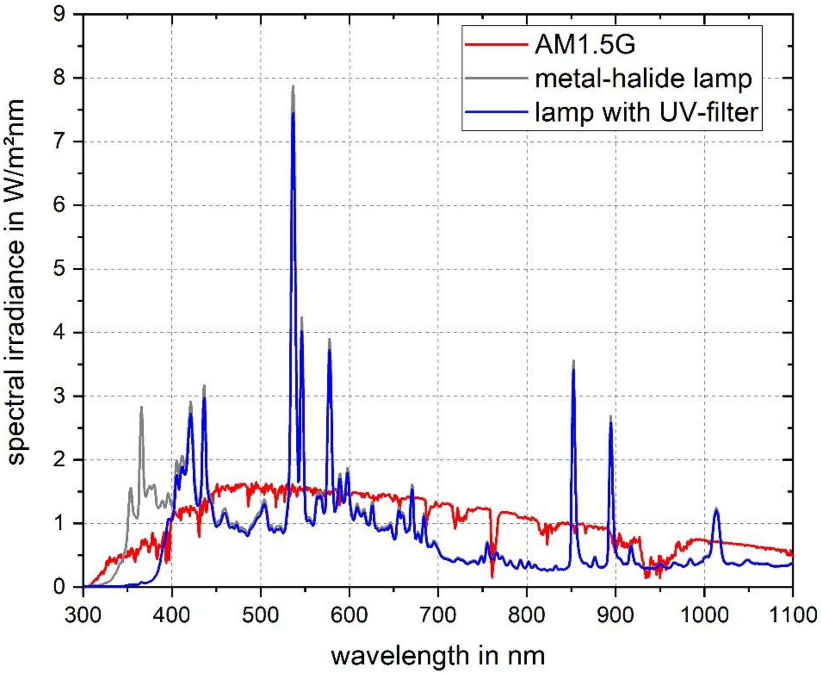

Fig. S24. Spectrum of the lamp of the High-throughput Ageing Setup used to age solar cells in comparison to AM1.5G. In this work, a UV filter was used (blue curve).

References

1. T. Dittrich and S. Fengler, "Surface Photovoltage Analysis of Photoactive Materials", World Scientific (Europe), 2020, DOI:10.1142/q0227.
2. I. Levine, A. Al Ashouri, A. Musiienko, *et al.* "Charge transfer rates and electron trapping at buried interfaces of perovskite solar cell", *Joule*, 2021, **5**, 2915–2933.
3. H. Köbler, S. Neubert, M. Jankovec, *et al.* "High-Throughput Aging System for Parallel Maximum Power Point Tracking of Perovskite Solar Cells", *Energy Technol.* 2022, **10**(6), 2200234
4. M.V. Khenkin, E.A. Katz, A. Abate, *et al.* "Consensus statement for stability assessment and reporting for perovskite photovoltaics based on ISOS procedures", *Nat. Energy,* 2020, **5**, 35–49.
5. L. Rakocevic, F. Ernst, N. T. Yimga, *et al.* "Reliable Performance Comparison of Perovskite Solar Cells Using Optimized Maximum Power Point Tracking", *Solar RRL,* 2019*,* **3**, 1800287.
6. T.M.Koh, V. Shanmugam, X.Guo, S. S. Lim, O. Filonik, E. M. Herzig, P. M.Buschbaum, V.Swamy, S. T.Chien, S. G. Mhaisalka, N. Mathews., " Enhancing moisture tolerance in efficient hybrid 3D/2D perovskite photovoltaics.", *J. Mater. Chem. A*, 2018, **6**, 2122-2128 (DOI. 10.1039/C7TA09657G).
7. H.Wang, C.Zhu, L. Liu, S. Ma, P. Liu, J. Wu, C.Shi,Q. Du, Y. Hao, S. Xiang, H. Chen, P. Chen, Y.Bai, H. Zhou, Y. Li, and Q. Chen., " Interfacial Residual Stress Relaxation in Perovskite Solar Cells with Improved Stability" *Adv. Mater.* 2019, **31**, 1904408
8. H. Kim, S.U. Lee, D. Y. Lee, *et al.*, "Optimal Interfacial Engineering with Different Length of Alkylammonium Halide for Efficient and Stable Perovskite Solar Cells", *Adv. Energy Mater.*, 2019, **9**, 1902740.
9. J.Han, K. Kim, J.Nam, S. J. Hong, E. J.Choi, D. Kim, In.Chung, H.S.Lin, T.-D. Kim, M. S.Strano, B. Han,J.W.Oh, H. D.Kim, and Il. Jeon., "Genetic Manipulation of M13 Bacteriophage for Enhancing the Efficiency of Virus-Inoculated Perovskite Solar Cells with a Certified Efficiency of 22.3%.", *Adv. Energy Mater.*,2021, **11**, 2101221
10. S. M. Yoon, H. Min, J. B. Kim, *et al.*, "Surface Engineering of Ambient-Air-Processed Cesium Lead Triiodide Layers for Efficient Solar Cells", *Joule*, 2021, 5, 183-196.
11. G. Huang, C. Wang, H. Zhang, S . Xu, Q. Xu, and Y. Cui., " Post-healing of defects: an alternative way for passivation of carbon-based mesoscopic perovskite solar cells via hydrophobic ligand Coordination.", *J. Mater. Chem. A*, 2018, **6**, 2449.
12. R. A. Belisle, K. A. Bush, L. Bertoluzzi, *et al.*, "Impact of Surfaces on Photoinduced Halide Segregation in Mixed-Halide Perovskites", *ACS Energy Lett.*, 2018, **3** (11), 2694-2700.
13. W. Meng, J. Xu, L. Dong, J. Zhang, Z. Xie, J. Luo, B. Zhao, K. Zhang, A. Osvet, T. Heumüller, K. Forberich, M.Halik, N. Li, C. J. Brabec., " An Innovative Anode Interface Combination for Perovskite Solar Cells with Improved Efficiency, Stability, and Reproducibility.", *Sol. RRL* 2022, **6**, 2200378.)
14. W. Li, X. Lai, F.Meng, G. Li, K. Wang, A.K. K.Kyaw, X.W. Sun., "Efficient defect-passivation and charge-transfer with interfacial organophosphorus ligand modification

for enhanced performance of perovskite solar cells.", *Solar Energy Materials and Solar Cells*, 2020, **211**, 110527.
15. T. H. Wu,.; G. D. Sharma, *et al*, "Surface Passivated Single Crystal Micro-Plates for Efficient Perovskite Solar Cells". *Processes* 2022, **10**, 1477.
16. W. Meng, Y. Hou, A. Karl, E. Gu, X. Tang, A. Osvet, K. Zhang, Y.Zhao, X.Du,J. G. Cerrillo, N.Li,,C. J. Brabec., " Visualizing and Suppressing NonradiativeLosses in High Open-Circuit Voltage *n-i-p*-Type $CsPbI_3$ Perovskite Solar Cells.", *ACS Energy Lett.* 2020, **5**, 271−279.
17. L. Canil, T. Cramer, B. Fraboni, *et al*., "Tuning Halide Perovskite Energy Levels", Energy Environ. Sci., 2021,14, 1429-1438.
18. N. K. Cho, H. J. Na, J. Yoo, *et al*. "Long-term stability in γ-$CsPbI_3$ perovskite via an ultraviolet-curable polymer network", *Commun. Mater.*, 2021 2, 30.
19. J. A. Steele, H. Jin, I. Dovgaliuk, *et al.* "Thermal unequilibrium of strained black $CsPbI_3$ thin films", *Science*, 2019, 365, 6454 679-684.
20. A. Marronnier, G. Roma, S. B. Richard, *et al.* "Anharmonicity and Disorder in the Black Phases of Cesium Lead Iodide Used for Stable Inorganic Perovskite Solar Cell", *ACS Nano*, 2018, 12, 4, 3477–3486.
21. Y. Wang, X. Liu, T. Zhang, *et al.* "The Role of Dimethylammonium Iodide in $CsPbI_3$ Perovskite Fabrication: Additive or Dopant?", *Angew. Chem.*, 2019, 131, 16844–16849.
22. J. Tauc, "Amorphous and Liquid Semiconductors.", Springer, Boston, MA, 1974, p. 159.
23. 11-J. Tauc., "Optical properties and electronic structure of amorphous Ge and Si.", *Materials Research Bulletin*, 1968, **3**,37-46.
24. E. V. Péan, S. Dimitrov, C. S. De Castro, *et al*., "Interpreting time-resolved photoluminescence of perovskite materials", *Phys. Chem. Chem. Phys.*, 2020, 22, 28345-28358
25. C. Stavrakas, A. A. Zhumekenov, R. Brenes, *et al*., "Probing buried recombination pathways in perovskite structures using 3D photoluminescence tomography", *Energy Environ. Sci.*, 2018, 11, 2846-2852
26. G. C. Xing, B. Wu, X. Y. Wu et al., "Transcending the slow bimolecular recombination in lead-halide perovskites for electroluminescence", *Nat. Comm.*, 2017, 8, 14558.
27. Q. Wang, E. Mosconi, C. Wolff, *et al.* "Rationalizing the Molecular Design of Hole-Selective Contacts to Improve Charge Extraction in Perovskite Solar Cells", *Adv. Energy Mater.*, 2019, 9, 1900990.
28. S. Fengler, T. Emmler, C. Wolpert, M. Schieda, M. Villa Vidaller, T. Klassen, et al., "Influence of Surface States and Mobility on Charge Transport in BiVO4 Investigated by Surface Photovoltage Spectroscopy." ECS Meeting Abstracts,2020, MA2020-01 I (39),1757, DOI: 10.1149/MA2020-01391756mtgabs
29. S. Fengler, H. Kriegel, M. Schieda, H. Gutzmann, T. Klassen, and T. Dittrich, "Defects Near c-Si(n++)/TiO2 Interfaces Revealed by Persistent Charging Analysis in Modulated Surface Photovoltage Spectroscopy." ECS Meeting Abstracts,2020, MA2020-01(39) ,1756, DOI: 10.1149/MA2020-01391756mtgabs

30. A. Musiienko, D. R. Ceratti, J. Pipek, *et al*., "Defects in Hybrid Perovskites: The Secret of Efficient Charge Transport", Adv. Funct. Mater., 2021, 31, 2104467.
31. Y. C. Lin, L. Y. Chen, & F. C. Chiu, "Lossy Mode Resonance-Based Glucose Sensor with High-κ Dielectric Film", *Cryst*., 2019, 9, 450.
32. F. Anwar, R. Mahbub, S. S. Satter, *et al*., "Effect of Different HTM Layers and Electrical Parameters on ZnO Nanorod-Based Lead-Free Perovskite Solar Cell for High-Efficiency Performance", *Int. J. Photoenergy,* 2017, DOI: 10.1155/2017/9846310.
33. A. Musiienko, J. Pipek, P. Praus, *et al*., "Deciphering the effect of traps on the electronic charge transport properties of methylammonium lead tribromide perovskites", *Sci. Adv*., 2020, **6**(37),eabb6393.
